# Iron homeostasis and macrophage polarization in oral squamous cell carcinoma: mechanisms and therapeutic perspectives

**DOI:** 10.3389/fimmu.2026.1863727

**Published:** 2026-07-29

**Authors:** Ahmed Bashah, Eslam Ghaleb, Ali Al-Waqeerah, Gang Chen

**Affiliations:** 1 First Affiliated Hospital of Dalian Medical University, Department of Stomatology, Dalian, China; 2 College of Basic Medical Science, Dalian Medical University, Dalian, China; 3 Department of Respiratory Medicine, The First Affiliated Hospital of Dalian Medical University, Dalian, China

**Keywords:** ferroptosis, immune checkpoint inhibitors, iron homeostasis, macrophage polarization, oral squamous cell carcinoma, tumor microenvironment, tumor-associated macrophages

## Abstract

Oral squamous cell carcinoma (OSCC) is an aggressive malignancy with a profoundly immunosuppressive tumor microenvironment and persistently poor responses to immune checkpoint inhibitors. Recent evidence highlights dysregulated iron metabolism as a critical yet underrecognized driver of this immunosuppressive state. Excess intratumoral iron perturbs macrophage polarization toward pro-tumoral M2-like phenotypes through iron-sensing pathways, including HIF-1α stabilization, IL-10/STAT6 signaling, and NF-κB-mediated redox programs, driving T-cell suppression, angiogenesis, and extracellular matrix remodeling that collectively reinforce resistance to PD-1/PD-L1 checkpoint blockade. Concurrently, iron-dependent regulated cell death via ferroptosis — governed by the GPX4–glutathione–*system Xc^-^* axis — represents an emerging immunological vulnerability, as ferroptotic tumor cells release damage-associated molecular patterns that potentiate anti-tumor CD8^+^ T-cell responses and synergize with checkpoint inhibitor activity. This review synthesizes current findings on the mechanistic interplay between iron homeostasis, macrophage phenotypic switching, and ferroptosis in OSCC and evaluates emerging therapeutic strategies targeting this axis, including iron chelators, ferroportin modulators, ferroptosis inducers, *Nrf2* pathway inhibitors, exosome-mimetic delivery systems, and iron-based nanoplatforms. While these approaches show promise, challenges remain regarding TME specificity, off-target iron depletion, and the absence of validated biomarkers for patient stratification. By integrating mechanistic insights with translational advances, this review underscores the therapeutic potential of targeting the iron–macrophage–ferroptosis axis and outlines how precision medicine-based interventions may overcome immune evasion and improve immunotherapy outcomes in OSCC.

## 1 Introduction

Oral squamous cell carcinoma (OSCC) is a malignant epithelial neoplasm arising from the mucosal lining of the oral cavity. It represents the predominant histologic subtype of oral cavity cancers, with > 90% of malignant oral tumors. It is also the most frequent form of head and neck squamous cell carcinoma, encompassing lesions from multiple intraoral sites, such as the tongue and floor of the mouth (1, 2). It is characterized by stepwise malignant transformation driven by accumulated genetic and epigenetic alterations in the oral epithelium, often influenced by carcinogens like smoking, alcohol abuse, and betel nut chewing, which are strongly associated with OSCC occurrence (3). Epidemiological studies reveal approximately 377,713 new lip and oral cavity cancer cases worldwide annually, with OSCC representing the predominant histologic subtype (4). Incidence and mortality are higher in men and in individuals over 50 years (5). Clinically, OSCC commonly presents as non-healing ulcers, leukoplakia, erythroplakia, or exophytic growths, which are frequently associated with discomfort, dysphagia, bleeding, and speech impairments. Advanced instances may manifest with regional lymphadenopathy and weight loss, which worsens the prognosis. Histopathological evaluation by biopsy remains the gold standard for diagnosis, providing information on tumor differentiation and depth of invasion (1, 6). Although biopsy is the gold standard for OSCC diagnosis, toluidine blue staining is the simplest, least intrusive, and highly accurate approach. Adjunctive tools, imaging contrast-enhanced CT and MRI, are used to determine tumor size and lymph node involvement, whilst PET scans can detect metastases (2). Despite advances in surgery, radiotherapy, and chemotherapy, the overall survival of patients with advanced OSCC remains poor, largely due to local invasion, regional lymph node metastasis (7, 8), and therapeutic resistance (9). Globally, OSCC continues to pose a significant health burden, emphasizing the urgent need for improved therapeutic strategies and a deeper understanding of tumor biology beyond cancer cell–intrinsic factors alone (10). The tumor microenvironment (TME) has emerged as a critical determinant of OSCC progression and treatment response. OSCC is characterized by a highly immunosuppressive microenvironment composed of tumor cells, immune cells, stromal components, and soluble mediators that collectively shape tumor evolution. Among immune cells, tumor-associated macrophages (TAMs) represent one of the most abundant and functionally influential populations within the OSCC TME (3, 11–14). Accumulating evidence indicates that TAM density and phenotype are closely associated with tumor aggressiveness, immune evasion, and poor clinical outcomes in OSCC (14–16).

In recent years, immune checkpoint inhibitors (ICIs), particularly antibodies targeting the programmed cell death protein 1 (PD-1) and its ligand PD-L1, have revolutionized the treatment landscape for recurrent and metastatic head and neck squamous cell carcinoma (HNSCC) (17–19), a category that includes OSCC (20). Landmark Phase III clinical trials, such as CheckMate-141 (19), and KEYNOTE-048, demonstrated overall survival benefits with nivolumab and pembrolizumab, respectively, establishing PD–1–based immunotherapy as a standard treatment option in the recurrent/metastatic setting (17, 18). However, only a subset of patients derive durable clinical benefit, while the majority exhibit primary or acquired resistance to ICIs. This highlights the presence of persistent immunosuppressive mechanisms within the OSCC tumor microenvironment (TME) and broader HNSCC TME that limit effective anti-tumor immune responses (20–22).

Macrophage polarization plays a central role in shaping the immunological tone of the TME. Macrophages exhibit remarkable plasticity and can adopt a spectrum of activation states, often simplified into pro-inflammatory, anti-tumoral M1-like macrophages and immunosuppressive, pro-tumoral M2-like macrophages. M2-polarized tumor-associated macrophages promote tumor growth by enhancing angiogenesis, extracellular matrix remodeling, epithelial–mesenchymal transition, and suppression of cytotoxic T-cell activity (23–25). In OSCC, increased infiltration of M2-like macrophages—particularly CD206^+^ cells, and in several studies also CD163^+^ cells—has been associated with advanced stage, lymph node metastasis, and unfavorable prognosis (23–26).

Parallel to immune dysregulation, altered iron metabolism has emerged as an important feature of many cancers. Iron is an essential micronutrient required for DNA synthesis, mitochondrial respiration, and cell proliferation; however, excess iron can drive oxidative stress and genomic instability (27). Cancer cells frequently exhibit an iron-addicted phenotype characterized by increased iron uptake, enhanced intracellular storage, and reduced iron export (27, 28). Dysregulated iron homeostasis has been implicated in tumor initiation, progression, and resistance to therapy across multiple cancer types (29). Systemic and cellular iron metabolism is tightly regulated by the hepcidin–ferroportin axis, transferrin receptors, and ferritin, and disruption of this balance in cancer contributes to an iron-rich tumor microenvironment that favors malignant growth (30, 31).

Importantly, iron metabolism and immune regulation are deeply interconnected (30, 31). Macrophages are central regulators of iron homeostasis, controlling iron storage, recycling, and release at both systemic and tissue levels (30). Emerging evidence demonstrates that macrophage polarization is closely linked to iron handling: pro-inflammatory M1-like macrophages tend to sequester iron, whereas alternatively activated M2-like macrophages more often display an iron-exporting phenotype that can support tumor progression (31). Experimental studies show that alterations in iron availability and heme/iron-handling pathways modulate macrophage polarization and inflammatory function, with iron restriction and HIF-1α induction in tumors driving immunosuppressive, M2-like TAMs, while iron overload and ROS can promote pro-inflammatory M1 phenotypes; the precise mechanisms and context-dependence of these iron-driven programs in tumor-promoting versus antitumor immunity are still being defined (31–35). Together, this iron–macrophage axis can create a feed-forward loop within the tumor microenvironment that favors angiogenesis and immunosuppression, thereby supporting tumor growth (30–32, 36).

In the context of OSCC, the convergence of dysregulated iron metabolism and TAM –mediated immunosuppression is increasingly recognized as a potential contributor to resistance to immune checkpoint inhibitors, although this mechanism remains under active investigation. Iron-rich TAM subsets have been shown to promote angiogenesis, dampen antigen presentation, and support an immunosuppressive tumor microenvironment, in part through reduced T-cell activation and increased production of inhibitory mediators (32). M2-like TAMs can express high levels of immunosuppressive cytokines such as IL-10 and TGF-β and upregulate immune checkpoint molecules, thereby limiting effective cytotoxic T-cell responses and potentially constraining the efficacy of immune checkpoint blockade. In parallel, recent studies linking iron-dependent cell death pathways such as ferroptosis with anti-tumor immunity and immunotherapy efficacy in head and neck squamous cell carcinoma and other cancers further underscore the relevance of iron metabolism in modulating responses to cancer immunotherapy (37, 38).

Given these observations, targeting iron homeostasis and macrophage polarization has emerged as a promising therapeutic strategy in cancer. Iron chelation, modulation of the hepcidin–ferroportin signaling axis (39), and approaches that reprogram tumor-associated macrophages toward anti-tumor phenotypes have shown potential to reshape the tumor microenvironment and enhance anti-tumor immunity in preclinical models. Combining such strategies with immune checkpoint inhibitors is increasingly being explored as a rational approach to overcoming resistance and improving outcomes (40). However, this concept remains largely supported by preclinical and early translational data and has yet to be validated in clinical trials in OSCC.

In this review, we comprehensively summarize current knowledge on iron homeostasis and macrophage polarization in OSCC, highlight their mechanistic interplay within the tumor microenvironment, and discuss therapeutic perspectives that aim to exploit the iron–macrophage axis to enhance immunotherapy responses.

## 2 Oral squamous cell carcinoma and the tumor microenvironment

### 2.1 Composition of the OSCC tumor microenvironment

**Figure 1:**
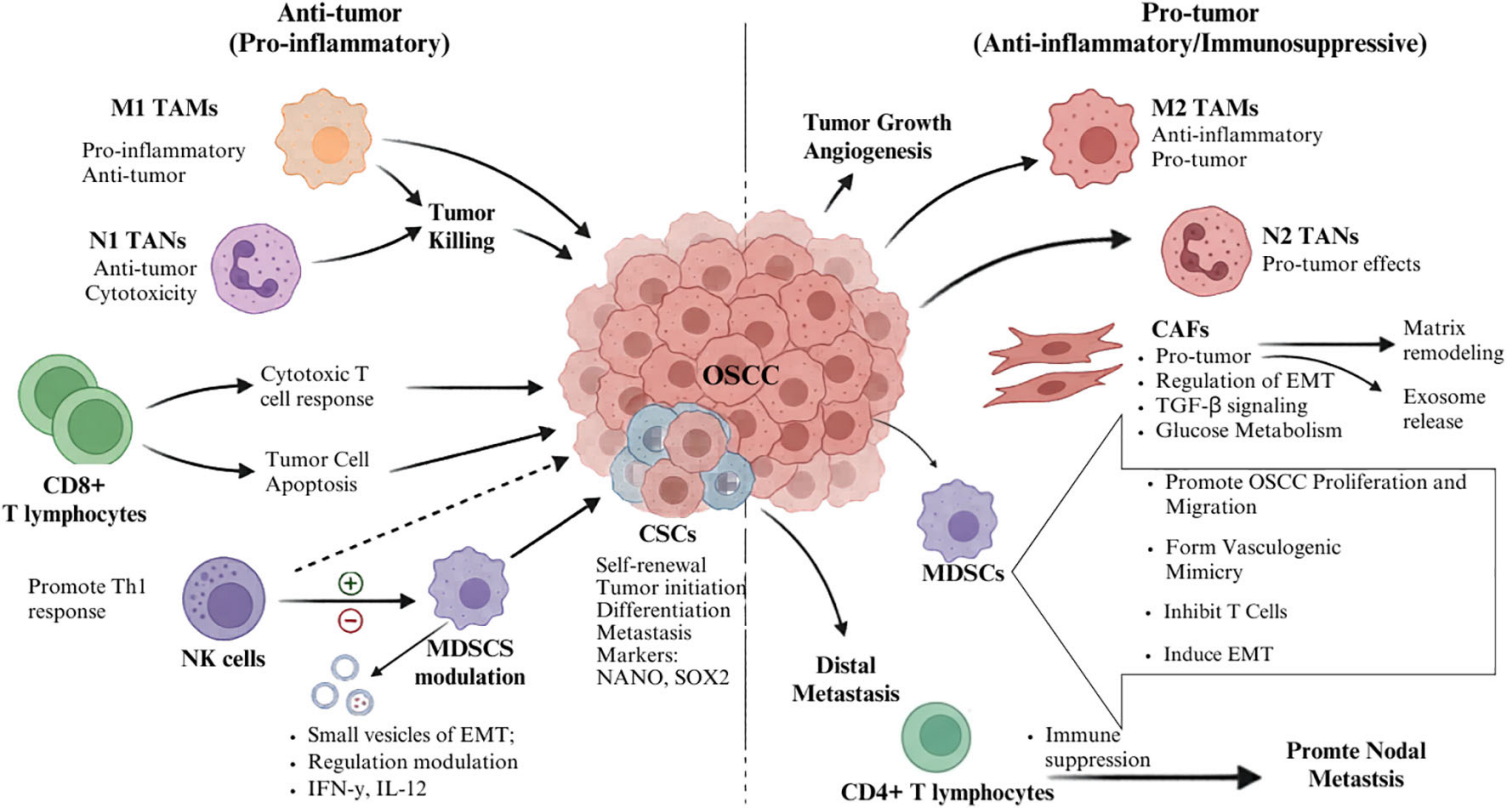
Schematic representation of the tumor microenvironment (TME) in OSCC, depicting principal cellular components including TAMs, CAFs, T lymphocytes, NK cells, MDSCs, and TANs.

TME of OSCC is a highly complex and dynamic network of cellular and non-cellular components that cooperate to influence tumor initiation, progression, and metastasis (3, 41). Various immune cells within the OSCC TME, including tumor-associated macrophages (TAMs), which often skew toward pro-tumoral, M2-like phenotypes, along with T lymphocytes (cytotoxic and regulatory T cells), natural killer (NK) cells, myeloid-derived suppressor cells (MDSCs), tumor-associated neutrophils (TANs), dendritic cells, cancer-associated fibroblasts (CAFs), and other myeloid subsets, constitute the major immune cell populations within the OSCC TME (3, 14, 23, 41). Endothelial cells are recruited during tumor-driven angiogenesis to establish a new vasculature that supplies oxygen and nutrients to the expanding tumor (3, 41) (**Figure 1**). Cancer-associated fibroblasts (CAFs) represent a major stromal population; they remodel the extracellular matrix (ECM) via enzymes such as matrix metalloproteinases and lysyl oxidase (LOX), thereby supporting invasion, angiogenesis, immunosuppression - through cytokines such as TGF-β, interleukin (IL) 6, and IL-10 - and treatment resistance (42–44).

The non-cellular compartment consists primarily of ECM proteins such as collagen, fibronectin, and laminin, together with a rich milieu of cytokines, chemokines, growth factors, extracellular vesicles carrying RNAs and proteins, and various metabolites that mediate crosstalk between tumor and stromal cells (3). Environmental factors, particularly hypoxia, further shape the OSCC TME by stabilizing hypoxia-inducible factors (HIFs) (45), inducing metabolic reprogramming, and promoting a more aggressive and therapy-resistant tumor phenotype (3, 12, 41).

### 2.2 Immunosuppressive nature of OSCC TME

The OSCC microenvironment is strongly immunosuppressive and employs multiple cellular and molecular mechanisms to facilitate immune evasion and protect tumor cells from effective immune surveillance. Tumor-associated macrophages, frequently enriched in OSCC and often polarized toward M2-like states, secrete anti-inflammatory cytokines such as interleukin-10 (IL-10) and transforming growth factor-β (TGF-β), which suppress effector T-cell and NK-cell functions and help establish an immune-privileged niche (3, 16, 23). These macrophages can also express high levels of programmed death-ligand 1 (PD-L1), contributing to T-cell exhaustion and immune escape via engagement of PD-1 on T cells (20, 23, 46, 47).

An imbalance between regulatory T cells (Tregs) and cytotoxic T lymphocytes (CTLs), with accumulation of Tregs and dysfunctional or exhausted CTLs, is frequently associated with an immunosuppressive milieu and unfavorable clinical outcomes in head and neck cancers, including OSCC (3, 43). Myeloid-derived suppressor cells (MDSCs) further reinforce this suppressive environment by inhibiting T-cell responses through production of reactive oxygen and nitrogen species, expression of arginase and other metabolic enzymes, and promotion of Treg expansion (48–50).

Tumor cells and stromal elements such as CAFs and TAMs can upregulate PD-L1 and modulate antigen-presentation pathways, reducing effective CTL-mediated recognition and contributing to immune escape (3, 20, 43). Hypoxia-inducible factors, stabilized under low-oxygen conditions in the TME, orchestrate broad metabolic and transcriptional changes that enhance angiogenesis, support the survival and suppressive function of TAMs and other myeloid cells, and promote resistance to radiotherapy and chemotherapy, thereby reinforcing the immunosuppressive and therapy-resistant state of OSCC (41, 45).

## 3 Iron metabolism in oral squamous cell carcinoma

### 3.1 The biological role of iron in oral cancer

The occurrence and development of OSCC are closely related to abnormal iron metabolism (37). Studies have shown that TFRC expression in OSCC tissues shows high regional heterogeneity — being significantly elevated at the invasive tumor front — mediating iron-loaded transferrin uptake and promoting increased cellular iron uptake, proliferation, and migration in tumor cells (51). Ferritin heavy chain 1 (FTH1) plays a central role in the regulation of iron homeostasis, and its overexpression in OSCC tissues is significantly associated with enhanced cell proliferation (52). In addition, OSCC cells generally exhibit “addiction” to iron - to support rapid proliferation, metastasis, and antioxidant defense, tumor cells upregulate iron uptake pathways and downregulate iron export by suppressing ferroportin activity through hepcidin-dependent signaling, which leads to intracellular iron accumulation (37, 53–55). It is worth noting that cancer stem cells (CSCs), as the culprit of tumor recurrence and metastasis, have a significantly higher dependence on iron and lipid metabolism than ordinary cancer cells across multiple cancer types (56), a principle that extends to OSCC.

### 3.2 The role of key molecules in iron metabolism in OSCC

Several studies have documented dysregulation of iron-handling proteins in OSCC (**Table 1**). Si et al. demonstrated that transferrin receptor 1 (*TFRC*) expression is significantly elevated at the invasive tumor front (ITF) of OSCC tumors compared with non-invasive areas, and that high *TFRC* expression correlates with increased cancer cell migration, proliferation, and poorer clinical outcomes in patients with aggressive invasion patterns. This effect was attributed to enhanced iron uptake via the transferrin (TF)–TFRC axis and was particularly pronounced in patients with high tumor-associated neutrophils and elevated *TFRC* expression (51). In a large OSCC cohort, Arora et al. reported that *TFRC* expression is significantly higher in tumor cells compared with most normal tissues, and that increased TFRC protein levels are associated with worse overall survival (OS), disease-specific survival (DSS), and progression-free interval PFI in HPV-negative OSCC patients. Their study also identified *TFRC* as one of two 3q22-3q29 amplified genes whose overexpression correlates with adverse clinical outcomes (57).

**Table 1:** Summary key molecules governing iron metabolism and ferroptosis sensitivity in oral squamous cell carcinoma (OSCC).

Protein/gene	Role	OSCC expression & regulation	Functional consequence in OSCC
TfR1 (*TFRC*)	Iron uptake receptor; pro-tumorigenic iron importer	Significantly overexpressed at invasive tumor front (ITF) compared to non-invasive OSCC areas; amplified at 3q22–3q29 locus; higher in OSCC tumor cells vs most normal tissues	High *TFRC* correlates with increased cancer cell migration and proliferation; it associates with worse OS and DSS in HPV-negative OSCC; amplification at 3q22–3q29 independently predicts adverse clinical outcomes; the effect is amplified in patients with high tumor-associated neutrophil infiltration
FTH1 (Ferritin Heavy Chain)	Intracellular iron storage protein; iron retention and redox buffering	Upregulated in OSCC tissue compared to adjacent non-tumor mucosa; overexpression reflects iron retention phenotype characteristic of iron-addicted tumor cells	FTH1 overexpression promotes OSCC cell proliferation and migration; reflects both iron sequestration and a tumor-promoting phenotype; elevated serum ferritin links to larger tumor size, lymph node metastasis, and local recurrence
Hepcidin (*HAMP*)/Ferroportin (FPN/*SLC40A1*)	Systemic iron regulator (hepcidin)/sole cellular iron exporter (ferroportin); hepcidin–FPN axis governs iron release	Direct OSCC-specific tissue data are limited; dysregulated hepcidin–ferroportin signaling is documented in the broader HNSCC context; hepcidin overexpression promotes ferroportin degradation and iron retention in epithelial malignancies	Hepcidin–FPN axis disruption drives iron sequestration in tumor cells, sustaining the iron-addicted phenotype; contributes to anemia of chronic disease in advanced OSCC; FPN overexpression in HNSCC cell lines induces cell-cycle arrest and senescence by depleting labile iron pools
GPX4 (Glutathione Peroxidase 4)	Master ferroptosis suppressor; reduces phospholipid hydroperoxides (PLOOH) using GSH	Overexpressed in cisplatin-resistant OSCC; constitutively elevated in OSCC cells with active Nrf2; regulated by FTO (m6A demethylase), PER1 overexpression, and Nrf2/HO-1 axis	High GPX4 confers simultaneous cisplatin resistance and ferroptosis resistance; GPX4 suppression by RSL3, artesunate, or carnosic acid restores ferroptotic sensitivity and reverses cisplatin resistance in OSCC; GPX4 inhibition is the primary mechanistic target of multiple ferroptosis-inducing agents active in OSCC
*SLC7A11* (xCT/System Xc^-^ subunit)	Ferroptosis suppressor; mediates cystine import for GSH biosynthesis; anti-oxidant metabolic gateway	Significantly overexpressed in OSCC tissue vs adjacent normal oral epithelium at both mRNA and protein levels; upregulated by EZH2/miR-125b-5p axis; suppressed by TCF12, miR-26a-5p, miR-34c-3p, and p53; circ_0000140 maintains high *SLC7A11* via miR-527 sponging	High *SLC7A11* expression independently predicts worse OS in TCGA OSCC cohorts; drives cisplatin resistance by sustaining GSH production and disabling ferroptosis; circ_0000140/miR-527/*SLC7A11* axis directly mediates DDP resistance in OSCC; sulfasalazine and erastin exploit *SLC7A11* dependence for therapeutic ferroptosis induction
FSP1 (Ferroptosis Suppressor Protein 1/AIFM2)	GPX4-independent ferroptosis suppressor; regenerates ubiquinol (CoQH_2_) as lipophilic radical scavenger	Expressed in OSCC; critically elevated in drug-tolerant persister (DTP) HNSCC subpopulations; FSP1-mediated CoQH_2_ recycling provides a secondary anti-ferroptotic defense when GPX4 is suppressed	FSP1 maintains ferroptosis resistance in therapy-refractory OSCC/HNSCC cells that survive initial treatment; FSP1 targeting selectively eliminates DTP cells and overcomes GPX4-inhibitor resistance; combined GPX4 + FSP1 inhibition required to fully suppress layered ferroptosis defense
Nrf2 (NFE2L2)	Master transcriptional regulator of anti-ferroptotic gene expression; drives *SLC7A11*, GPX4, HO-1, and ferritin	Constitutively activated in OSCC via KEAP1 inactivation, p62/SQSTM1-mediated KEAP1 sequestration, or epigenetic silencing; sustains simultaneous overexpression of multiple ferroptosis suppressors; NFE2L1 (NRF1) also active in OSCC	Constitutive Nrf2 activation creates broad, coordinated ferroptosis and cisplatin resistance; Nrf2 inhibition by artesunate, carnosic acid, or brusatol restores sensitivity to ferroptosis inducers and platinum chemotherapy; Nrf2/HO-1 pathway is the dominant resistance axis in cisplatin-resistant OSCC
TPI1 (Triosephosphate Isomerase 1)	Glycolytic enzyme; metabolic link between glucose metabolism and ferroptosis resistance	Overexpressed in OSCC; contributes to cisplatin resistance through metabolic mechanisms involving NADPH regeneration and altered redox balance; promotes OSCC progression	TPI1 overexpression promotes OSCC cell growth while mediating resistance to ferroptosis; TPI1 silencing sensitizes cisplatin-resistant OSCC cells to ferroptotic stimuli by disrupting NADPH-dependent antioxidant regeneration; links glycolytic reprogramming to ferroptosis suppression
*NSUN2* (NOP2/Sun RNA Methyltransferase 2)	m5C RNA methyltransferase; epigenetic regulator of ferroptosis resistance	Overexpressed in OSCC; mediates N5-methylcytosine (m5C) modification of target mRNAs that regulate ferroptosis pathway components; represents an epigenetic layer of ferroptosis regulation distinct from genetic alterations	*NSUN2* overexpression enhances ferroptosis resistance in OSCC by inhibiting ferroptotic cell death through m5C-mediated post-transcriptional regulation; *NSUN2* is a novel epigenetic target whose inhibition may reverse ferroptosis resistance in OSCC without directly targeting the GSH–GPX4 axis
p53 (TP53)	Context-dependent ferroptosis modulator; suppresses *SLC7A11* transcription; also activates p21/CDKN1A to partially restrain ferroptosis	Frequently mutated or functionally inactivated in tobacco/alcohol-associated OSCC; wild-type p53 transcriptionally suppresses *SLC7A11* and promotes ferroptotic sensitivity; PER1 circadian gene functions as a p53 effector regulating HIF-1α/*SLC7A11* in OSCC	p53 loss derepresses *SLC7A11* → elevated GSH → ferroptosis resistance in OSCC; resveratrol-mediated p53/*SLC7A11* axis restoration re-sensitizes OSCC to ferroptosis; PER1 overexpression promotes ferroptosis through HIF-1α degradation and downstream *SLC7A11*/GPX4 suppression; p53 mutation status must inform ferroptosis-based treatment strategy
ACSL4/LPCAT3	Pro-ferroptotic metabolic enzymes; incorporate PUFAs into membrane phosphatidylethanolamines (PE) — the ferroptosis substrate	ACSL4 upregulated upon CK19 silencing in OSCC; activated by YAP/TAZ phase separation in manganese-stimulated OSCC model; LPCAT3 co-regulates PUFA-PE pool size; together determine ferroptosis substrate availability	ACSL4/LPCAT3 upregulation renders OSCC membranes rich in oxidizable PUFA-PE, increasing ferroptosis susceptibility; ACSL4 elevation is a molecular switch that sensitizes resistant OSCC subpopulations to iron-dependent lipid peroxidation; ACSL4 expression is a potential stratification biomarker for ferroptosis-based therapy
STARD4-AS1/circ_0000140 (lncRNA/circRNA regulators)	Non-coding RNA ferroptosis modulators; epigenetic/post-transcriptional regulation of ferroptosis sensitivity	STARD4-AS1 identified as novel ferroptosis-related lncRNA biomarker in OSCC by bioinformatic analysis; circ_0000140 overexpressed in cisplatin-resistant OSCC cell lines and acts as miR-527 sponge to maintain *SLC7A11* expression	High circ_0000140 → suppressed miR-527 → elevated *SLC7A11* → ferroptosis and cisplatin resistance; silencing circ_0000140 restores ferroptotic death and DDP sensitivity in resistant OSCC; STARD4-AS1 represents a transcriptomic prognostic biomarker linking ferroptosis gene expression to OSCC clinical outcomes

TfR1/*TFRC*, transferrin receptor 1; FTH1, ferritin heavy chain 1; FPN/*SLC40A1*, ferroportin; GPX4, glutathione peroxidase 4; *SLC7A11*/xCT, solute carrier family 7 member 11; FSP1/AIFM2, ferroptosis suppressor protein 1; Nrf2/NFE2L2, nuclear factor erythroid 2-related factor 2; TPI1, triosephosphate isomerase 1; NSUN2, NOP2/Sun RNA methyltransferase 2; ACSL4, acyl-CoA synthetase long-chain family member 4; LPCAT3, lysophosphatidylcholine acyltransferase 3; HNSCC, head and neck squamous cell carcinoma; OS, overall survival; DSS, disease-specific survival; ITF, invasive tumor front; GSH, glutathione; PLOOH, phospholipid hydroperoxides; CoQH_2_, ubiquinol; DTP, drug-tolerant persister; DDP, cisplatin; LIP, labile iron pool; PUFA-PE, polyunsaturated fatty acid phosphatidylethanolamine; m5C, 5-methylcytosine.

Ferritin heavy chain 1 (FTH1), the major intracellular iron storage protein, has also been implicated in OSCC. Huang et al. showed that *FTH1* is upregulated in OSCC tissue and that ferritin overexpression promotes proliferation and migration of OSCC cells. This suggests that increased ferritin expression reflects both iron retention and a tumor-promoting phenotype in OSCC (52). Although there is still little literature on hepcidin expression in OSCC specifically, dysregulated hepcidin signaling is a recurring motif in epithelial malignancies, where hepcidin overexpression promotes iron retention and leads to abnormal iron metabolism. Hepcidin-ferroportin axis disruptions are directly documented in HNSCC: ferroportin overexpression in metastatic HNSCC cell lines (HN12, JHU-022) depletes the labile iron pool and induces cell cycle arrest and senescence, effects that are reversed by hepcidin treatment— confirming that hepcidin-driven ferroportin internalization actively sustains iron retention and proliferation in HNSCC (37, 55). OSCC cells exhibit constitutive overactivation of anti-ferroptotic mechanisms, including GPX4, SLC7A11, FSP1, and Nrf2, and experimental disruption of these axes has been shown to suppress tumor growth while restoring sensitivity to multiple treatment modalities (53). Beyond these canonical nodes, triosephosphate isomerase 1 (TPI1) promotes OSCC progression by regulating glucose metabolism and mediating resistance to ferroptosis (58). The m5C methyltransferase NSUN2 is overexpressed in OSCC, enhancing ferroptosis resistance through m5C methylation of SQSTM1/P62 mRNA, thereby stabilizing it via the reader protein YBX1 to suppress autophagy-dependent ferroptosis (59).

Genetic and epigenetic regulators, including p53, PER1, circ_0000140, and STARD4-AS1, critically modulate ferroptotic sensitivity, while metabolic enzymes such as ACSL4, LPCAT3, and TPI1 link ferroptosis to cellular plasticity and resistance. Preclinical studies highlight the promise of small-molecule inhibitors, repurposed agents (e.g., sorafenib, artesunate, trifluoperazine), natural compounds (e.g., piperlongumine, Evodia lepta, quercetin), and nanomedicine platforms for targeted ferroptosis induction. Within the tumor microenvironment, ferroptosis exerts immunogenic and context-dependent dual roles, and genomic and transcriptomic evidence links ferroptosis-related genes to patient prognosis. Beyond cancer, ferroptosis also contributes to non-malignant oral diseases, including pulpitis, periodontitis, and infection-associated inflammation, where inhibitors may protect tissues. Despite these advances, clinical translation is constrained by the lack of safe ferroptosis inducers and validated biomarkers; future research should focus on developing pharmacologically viable GPX4 inhibitors, refining biomarker-driven patient stratification, and designing multimodal regimens that combine ferroptosis induction with standard therapies while preserving immune and tissue integrity (53). These findings establish that key molecules governing iron metabolism and ferroptosis sensitivity are intimately linked to OSCC progression, therapeutic resistance, and clinical outcomes.

### 3.3 Clinical association between iron metabolism disorders and OSCC

Clinical evidence supports a role for iron metabolism dysregulation in OSCC progression and prognosis. Arora et al. showed that high *TFRC* protein levels in OSCC tissue microarrays are independently associated with worse overall survival, disease-specific survival, and progression-free interval, even after adjustment for pathological stage and other clinicopathologic variables, underscoring its value as an adverse prognostic biomarker (57). Si et al. observed that OSCC patients with high *TFRC* expression at invasive tumor fronts had worse survival outcomes and more aggressive invasion patterns than those with low *TFRC* expression, indicating that iron uptake mechanisms contribute to tumor aggressiveness and regional progression (51). Elevated ferritin expression in OSCC and related HNSCC tumors has been associated with metastatic potential and worse prognosis, consistent with a role for enhanced iron storage in supporting tumor advancement and resistance to cell stress. Combined, these findings indicate that altered iron uptake and storage mechanisms in OSCC correlate with higher tumor stage, lymph node metastasis, and poor clinical outcomes (37, 52, 60). Beyond tissue-based evidence, systemic indicators of iron metabolism further reflect disease burden. Anemia is present in 56–88% of patients with OSCC, and the degree of anemia is positively correlated with tumor progression. Additionally, serum iron levels show a weak correlation with hemoglobin, suggesting competitive iron consumption between the tumor and bone marrow (61). Notably, serum ferritin in OSCC patients (162.47 ng/mL) is significantly higher than in precancerous lesions (62.70 ng/mL), and ferritin levels decrease markedly six months after treatment, positioning it as a potential prognostic marker (60). Additionally, abnormal serum concentrations of trace elements such as iron, copper, and zinc in OSCC patients are related to disease occurrence and betel nut chewing habits (62). These clinical associations underscore that iron metabolism disorders—manifesting both locally within tumors and systemically in serum—are intimately linked to OSCC aggressiveness, prognosis, and modifiable risk factors.

### 3.4 The potential of ferroptosis in the treatment of OSCC

Based on the core role of iron metabolism in OSCC, multiple precise intervention strategies are being explored, ranging from small-molecule inducers and key target inhibitors to nanomedicine platforms and combination regimens. System Xc^-^ inhibitors such as erastin and sulfasalazine block cystine uptake, leading to glutathione (GSH) depletion and impaired GPX4 activity, thereby inducing ferroptosis and restoring cisplatin sensitivity in OSCC models (53, 63). Direct GPX4 inhibitors (64), including RSL3 and FIN56, trigger lethal lipid peroxidation and have demonstrated ferroptotic cell death in CAL27, SCC9, and HSC3 cells (53). Iron modulators that activate ferritinophagy increase labile iron pools and lipid ROS, further enhancing ferroptosis (65). Small-molecule compounds with diverse mechanisms include resveratrol, which activates p53 and represses *SLC7A11* to reduce GSH while increasing Fe²^+^ and ROS, inhibiting OSCC proliferation and invasion (66, 67); quercetin, which inactivates the mTOR/S6K pathway to induce ferroptosis and enhance cisplatin efficacy (68); manganese ions, which trigger ferroptosis via YAP/TAZ phase separation-mediated ACSL4 activation and subsequent lipid peroxidation in OSCC (69). Repurposed drugs have shown promise: artesunate induces iron-dependent ROS and lipid peroxidation, synergizing with cisplatin to inhibit OSCC (70, 71); Sorafenib, through its off-target inhibition of System Xc^-^ (SLC7A11/xCT), blocks cystine uptake and depletes GSH to induce ferroptosis, and has been explored in combination with cisplatin to overcome chemoresistance (70, 72–74); trifluoperazine (TFP) inhibits GPX4 and induces autophagic ferroptosis, with poor prognosis linked to high *GPX4* expression (75);quisinostat (HDACi) promotes ROS stress and lipid peroxidation, sensitizing OSCC to ferroptosis (76); disulfiram (with or without copper) suppresses the Nrf2/HO-1 axis, thereby promoting ferroptosis (77), and melatonin amplifies ROS and mitochondrial stress, potentiating erastin-induced ferroptosis (78).

Natural compounds provide additional options: piperlongumine downregulates *GPX4*/*SLC7A11* and increases ROS, suppressing growth and synergizing with CB-839 (79); *Evodia lepta* extract reduces GPX4/HSPA5 and PD-L1, exerting cytotoxic and immunomodulatory effects (80); brusatol inhibits Nrf2/GCLC, lowers SLC7A11, and depletes GSH, promoting ferroptosis and suppressing OSCC growth *in vitro* and *in vivo* (81); fucoxanthin downregulates *Nrf2/HO-1/GPX4* while increasing ROS, Fe²^+^, and p53, inducing ferroptosis in SCC-25 tongue carcinoma cells (82); baicalin suppresses ferritin heavy chain 1 (FTH1), inhibits epithelial-mesenchymal transition (EMT), and enhances ferroptosis to reduce proliferation and invasion (83); and Ganoderma lucidum spore powder (A-GSP) increases Fe²^+^ influx, depletes GSH, upregulates *ACSL4*, downregulates *GPX4*, and induces mitochondrial dysfunction, suppressing OSCC growth *in vivo* (84). Nanotechnology-based approaches offer additional platforms: DMEFe nanoparticles combined with chemotherapy and ferroptosis induction have been applied in OSCC (85); iron oxide and mesoporous silica-based nanoreactors integrate photodynamic and chemical kinetic therapies (86, 87); NK cell exosome-encapsulated gold-manganese nanoclusters (AMNCs) target enhanced ferroptosis (88); carbon dot–hydrogel films enable theranostic Fe³^+^ detection coupled with ferroptosis (89); Fe-dopamine composites generate Fenton-like ROS to induce lipid peroxidation (90);sorafenib-Ce6 nanoparticles combine photodynamic therapy with ferroptosis to overcome hypoxia resistance (74), exosome-encapsulated gold-manganese (Exo-AuMn) nanoclusters generate ROS and enable immune-targeted selective ferroptosis with imaging capabilities (88); and CD44-targeted mP6/Rg3 micelles inhibit ABCB1 and promote ferroptosis in cancer stem cells, suppressing CSC proliferation, migration, and OSCC growth *in vitro* and *in vivo* (91). Combination therapy optimization has been extensively explored. Chemo-ferroptosis synergy is achieved by combining cisplatin with ferroptosis inducers: erastin (System Xc^-^ inhibitor) and RSL3 (GPX4 inhibitor), both of which restore lipid ROS and ferroptotic cell death in cisplatin-resistant OSCC cells *in vitro* and *in vivo*, representing a promising strategy to overcome chemoresistance (53, 92, 93); *Evodia lepta* suppresses *GPX4/HSPA5*, contrasting cisplatin’s upregulation, suggesting utility in cisplatin-resistant OTSCC (80); carnosic acid plus cisplatin inactivates Nrf2/HO-1 to reverse cisplatin resistance (94); amoxicillin plus cisplatin induces mitochondrial dysfunction and ferroptosis, enhancing cisplatin efficacy (95), and RSL3 combined with LYN-1604 triggers synergistic autophagy-ferroptosis tumor suppression (96).

**Figure 2:**
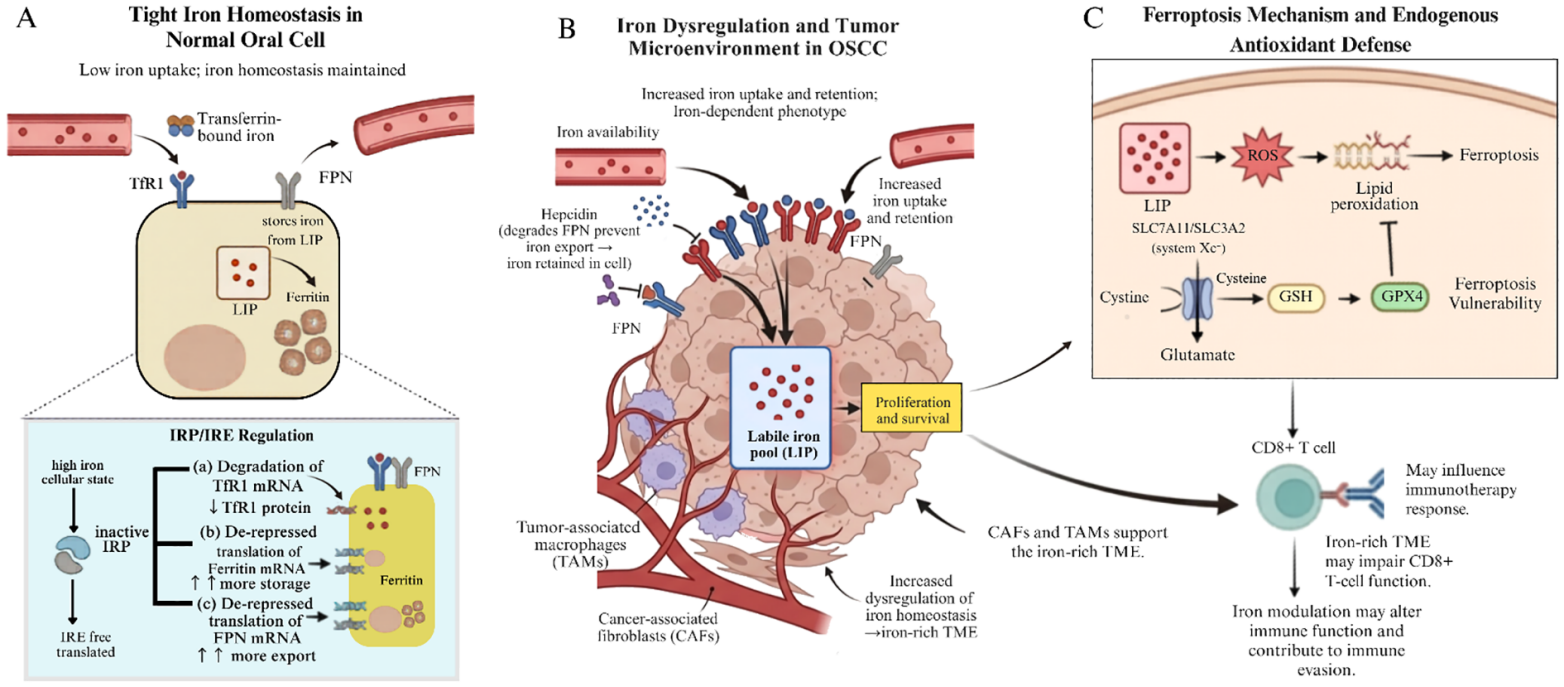
Iron homeostasis in normal oral epithelium, dysregulated iron metabolism in the OSCC tumor microenvironment, and the ferroptosis pathway as a therapeutic interface. **(A)** Normal oral epithelial cells maintain iron homeostasis through a tightly regulated balance between transferrin-bound iron uptake via TfR1, intracellular buffering within the labile iron pool (LIP), ferritin-mediated iron storage (sequestered from the LIP), and export via ferroportin (FPN). Under iron-sufficient conditions, IRP inactivation coordinately: (a) destabilizes TfR1 mRNA, reducing iron uptake; (b) de-represses ferritin mRNA translation, increasing iron storage; and (c) de-represses FPN mRNA translation, promoting iron export — collectively preventing intracellular iron excess. **(B)** In OSCC, the iron-addicted tumor phenotype is driven by TfR1 upregulation and hepcidin-mediated FPN degradation, which together markedly increase iron uptake and block export, expanding the labile iron pool (LIP) to support tumor proliferation and survival. Tumor-associated macrophages (TAMs) and cancer-associated fibroblasts (CAFs) further enrich the iron content of the tumor microenvironment, establishing a self-reinforcing oncogenic iron cycle. **(C)** The expanded LIP drives Fenton-type ROS generation, initiating lipid peroxidation and culminating in ferroptotic cell death. system Xc^-^ (SLC7A11/SLC3A2) imports cystine, which is reduced to cysteine for GSH biosynthesis, enabling GPX4-mediated reduction of phospholipid hydroperoxides; disruption of either axis increases ferroptosis vulnerability. The iron-rich TME also impairs CD8^+^ T-cell function and may influence responses to immune checkpoint inhibitor therapy. TfR1, transferrin receptor 1; FPN, ferroportin; LIP, labile iron pool; IRP, iron regulatory protein; IRE, iron-responsive element; *SLC7A11*, solute carrier family 7 member 11; GSH, glutathione; GPX4, glutathione peroxidase 4; ROS, reactive oxygen species; TAMs, tumor-associated macrophages; CAFs, cancer-associated fibroblasts; OSCC, oral squamous cell carcinoma.

Ferroptosis inducers such as statins sensitize radioresistant HPV-negative HNSCC to radiotherapy by inducing ferroptotic cell death (97); hyperbaric oxygen plus ionizing radiation suppresses GPX4, enhances ferroptosis, and re-sensitizes radio-resistant OSCC cells, improving tumor control (98). Finally, immunotherapy combined with ferroptosis downregulates PD-L1 and activates immune responses, potentially enhancing immune checkpoint inhibitor efficacy (38, 80). In parallel, resistance mechanisms are being characterized: In cadmium-exposed oral cancer cells, cadmium induces ferroptosis by activating NCOA4-mediated ferritinophagy, but MT2A overexpression mediates resistance (65). A range of pharmacological agents targeting the ferroptosis pathway have been identified as therapeutically relevant in OSCC, and their principal mechanisms and combination prospects are summarised in **Table 2**. The convergence of disrupted iron homeostasis, dysregulated ferroptosis pathway, and immune microenvironment modulation in OSCC is depicted schematically in **Figure 2**.

**Table 2:** Pharmacological agents targeting the ferroptosis pathway in oral squamous cell carcinoma.

Agent	Class	Primary target	Mechanism of action in OSCC	Combination potential and translational notes
Erastin	Small-molecule inhibitor	*SLC7A11*/System Xc^-^	Erastin blocks cystine import through the *SLC7A11* transporter, resulting in glutathione depletion and subsequent inactivation of glutathione peroxidase 4 (GPX4). The resulting impairment of phospholipid hydroperoxide (PLOOH) detoxification drives accumulation of lipid peroxides and ultimately ferroptotic cell death.	Demonstrated synergy with cisplatin in OSCC cell models. A combined regimen with sulfasalazine and an anti-interleukin-1β monoclonal antibody has been reported to enhance ferroptotic killing alongside immune modulation. Co-administration with melatonin has also been explored to augment ferroptosis in oral cancer.
RSL3	Small-molecule inhibitor	GPX4 (direct covalent inhibitor)	RSL3 directly and covalently inactivates GPX4, abrogating the enzymatic detoxification of phospholipid hydroperoxides. This loss of PLOOH clearance leads to unrestricted lipid peroxidation and disruption of membrane integrity. Notably, RSL3 has demonstrated selective cytotoxicity against OSCC cells while sparing normal oral fibroblasts, supporting a degree of therapeutic index.	Combined use with cetuximab has been proposed to overcome concurrent EGFR and ferroptosis resistance in head and neck squamous cell carcinoma (HNSCC). Synergy with cisplatin has been documented in OSCC models. Co-application with Nrf2 pathway inhibitors, including artesunate and carnosic acid, further amplifies ferroptotic cell death.
Sulfasalazine	Approved anti-inflammatory agent (repurposed)	*SLC7A11*/System Xc^-^	Sulfasalazine inhibits the SLC7A11 subunit of the cystine/glutamate antiporter, reducing intracellular cystine availability and depleting glutathione stores. In addition to inducing ferroptosis via this pathway, sulfasalazine has been shown to modulate interleukin-1β production within the OSCC tumor microenvironment, thereby combining direct cytotoxic and immunomodulatory mechanisms.	When co-administered with an anti-interleukin-1β monoclonal antibody in OSCC, the combination produces enhanced ferroptosis alongside modulation of the inflammatory tumor microenvironment. Its established clinical safety record in inflammatory conditions makes it a plausible candidate for drug repurposing.
Artesunate	Artemisinin derivative (repurposed antimalarial)	Reactive oxygen species (ROS) generation; Nrf2 pathway inhibition	Artesunate exploits the characteristically elevated labile iron pool (LIP) present in OSCC cells to catalyze Fenton-type reactions, generating reactive oxygen species that initiate lipid peroxidation. Concurrent inhibition of the Nrf2 antioxidant pathway amplifies ferroptotic vulnerability by suppressing compensatory antioxidant responses that would otherwise limit cell death.	The combination of artesunate with Nrf2 inhibitors has been reported to reverse cisplatin resistance in HNSCC models. Synergistic activity has also been noted with histone deacetylase inhibitors, including quisinostat, in squamous tumor models, suggesting potential for multi-pathway combination protocols.
Trifluoperazine (TFP)	Repurposed antipsychotic agent	GPX4; *SLC7A11*; autophagy regulation	Trifluoperazine inhibits GPX4 activity and elevates intracellular lipid reactive oxygen species, while simultaneously promoting autophagy-dependent ferroptosis through modulation of the *SLC7A11*/GPX4 signaling axis in OSCC cells. The compound therefore acts through convergent mechanisms linking ferroptosis induction to autophagic cell death pathways.	As a clinically established drug with a well-characterized safety profile in psychiatry, trifluoperazine represents a drug repurposing candidate. Potential synergy with reactive oxygen species-amplifying agents has been proposed, and its existing clinical approval may facilitate translational investigation in oral cancer.
Brusatol	Natural quassinoid (plant-derived)	Nrf2/GCLC pathway	Brusatol inhibits the Nrf2 transcription factor and its downstream target glutamate-cysteine ligase catalytic subunit (GCLC), thereby suppressing *SLC7A11* expression and reducing glutathione biosynthesis. The resulting depletion of glutathione promotes ferrous iron accumulation and reactive oxygen species generation, ultimately inducing ferroptosis. This mechanism has been demonstrated in OSCC CAL-27 cells.	Brusatol is particularly relevant to OSCC given the constitutive Nrf2 activation observed in many oral cancers, which confers resistance to oxidative stress-based therapies. Combination with GPX4 inhibitors is anticipated to produce additive ferroptosis induction. Oral bioavailability and systemic tolerability require further pharmacological evaluation.
Carnosic acid	Natural rosemary polyphenol	Nrf2/HO-1 pathway	Carnosic acid inactivates the Nrf2/heme oxygenase-1 (HO-1) antioxidant axis, thereby reducing cellular capacity to neutralize lipid peroxides and enhancing ferroptotic susceptibility. This mechanism sensitizes OSCC cells to cisplatin-induced ferroptosis by removing a key cytoprotective barrier that would otherwise limit lipid oxidation-driven cell death.	Cisplatin sensitization in OSCC has been demonstrated, and the compound’s derivation from natural sources confers a generally favorable safety profile. Carnosic acid is considered a candidate for integration into combined oral chemo-ferroptosis therapeutic protocols, although clinical pharmacokinetic data in oncology settings are limited.
Iron oxide nanoparticles (IONPs; including ferumoxytol)	Nanomaterial/Iron-based therapeutic platform	Labile iron pool (LIP) expansion; Fenton chemistry; tumor-associated macrophage (TAM) reprogramming	Iron oxide nanoparticles release catalytic ferrous iron intracellularly following uptake, which participates in Fenton-type reactions to generate reactive oxygen species and initiate lipid peroxidation. In addition, the iron released from IONPs reprograms tumor-associated macrophages from an immunosuppressive M2 phenotype toward a pro-inflammatory M1 phenotype, combining direct ferroptotic cytotoxicity with immunological remodeling of the tumor microenvironment. Mannose-modified IONPs can be selectively delivered to CD206-expressing M2 macrophages.	Ferumoxytol is a clinically approved intravenous iron formulation that has demonstrated preclinical tumor growth inhibition via macrophage reprogramming. Combination with radiotherapy to amplify radiation-induced ferroptosis in OSCC has been explored. Mannose-modified nanoparticle platforms for targeted CD206^+^ TAM delivery represent an advanced direction for tumor microenvironment-directed therapy.
Sorafenib	Multi-kinase inhibitor (approved for hepatocellular and renal cell carcinoma)	System Xc^-^/*SLC7A11*; GSH pathway (secondary mechanism)	In addition to its canonical receptor tyrosine kinase inhibition, sorafenib induces ferroptosis through partial suppression of *SLC7A11* expression and reduction of intracellular glutathione stores, thereby sensitizing cancer cells to lipid peroxidation-driven cell death. This ferroptosis-inducing capacity has been harnessed in photodynamic co-delivery platforms in oral cancer models.	A carrier-free nanoparticle platform co-delivering sorafenib with the photosensitizer chlorin e6 (Ce6) demonstrated synergistic potentiation of photodynamic therapy and ferroptosis in oral cancer. Drug repositioning for OSCC is under investigation, given the established clinical pharmacology of sorafenib in other solid tumor indications.

LIP, labile iron pool; GPX4, glutathione peroxidase 4; *SLC7A11*, solute carrier family 7 member 11; GSH, glutathione; PLOOH, phospholipid hydroperoxides; ROS, reactive oxygen species; Nrf2, nuclear factor erythroid 2-related factor 2; HO-1, heme oxygenase-1; GCLC, glutamate-cysteine ligase catalytic subunit; TAM, tumor-associated macrophage; PDT, photodynamic therapy; Ce6, chlorin e6; ZVI, zero-valent iron; HNSCC, head and neck squamous cell carcinoma; OSCC, oral squamous cell carcinoma; EGFR, epidermal growth factor receptor.

### 3.5 Microenvironment and cross-dimensional interaction

The tumor microenvironment in OSCC contributes to the regulation of ferroptosis through multiple cross-dimensional interactions. Iron accumulation indirectly promotes radiotherapy resistance by regulating immune cell function and oxidative stress levels in TME. Glutamine inhibition combined with CD47 blockade has been shown to enhance radiotherapy-induced ferroptosis in HNSCC by increasing labile iron pool availability and amplifying lipid peroxidation, providing a combination strategy to overcome radioresistance (99). Regarding the microbial niche, OSCC-related oral flora acts as an iron ion transport that promotes tumor growth by upregulating genes involved in iron transport and haemolysins (100). Within the immune compartment, ferroptosis exhibits immunogenic properties and may reshape the OSCC tumor microenvironment, potentially converting immunologically cold tumors into hot ones responsive to immunotherapy (53). Furthermore, the extracellular matrix plays a role: Plasma-activated Ringer’s lactate solution (PAL) selectively induces ferroptosis in OSCC cells and suppresses migration and invasion, with parallel downregulation of lysyl oxidase (LOX) and reduction in collagen formation as downstream effects (101). In summary, ferroptosis modulation in OSCC is not cell-autonomous but rather emerges from dynamic interplay among oral microbiota, immune cells, and structural matrix components.

### 3.6 Clinical transformation and prospects

Several avenues are emerging to translate ferroptosis biology into clinical practice for OSCC. Prognostic markers based on ferroptosis-related genes (FRGs) can predict overall survival in OSCC patients (102); notably, high *TFRC* expression is associated with poor prognosis (51). In terms of therapeutic innovation, combination therapy employing ferroptosis-inducing agents together with standard chemotherapy or immunotherapy represents a new direction to overcome drug resistance. However, critical challenges remain. The dual role of ferroptosis—particularly its selectivity between tumor and non-tumor tissues, along with its microenvironment-dependent effects—needs to be carefully addressed before clinical application (53).

## 4 Macrophage polarization: concepts and mechanisms

### 4.1 M1 vs M2 macrophages

Macrophages are polarized into M1 and M2 phenotypes, with M1 having anti-tumoral effects and M2 having pro-tumoral effects; M2 macrophages are further subdivided into four subtypes according to different microenvironmental stimuli (23). The polarization of macrophages represents a functional continuum rather than a binary state, determined by the local cytokine environment. Classically activated M1 macrophages, stimulated by IFN-γ and LPS, are characterized by the secretion of pro-inflammatory cytokines (IL-6, IL-12, IL-1β, TNF-α) and high expression of MHC II, CD80, and CD86 (103, 104). In contrast, alternatively activated M2 macrophages, driven by IL-4 and IL-13, upregulate scavenger receptors such as CD206 and secrete anti-inflammatory mediators (IL-10, TGF-β, CCL17) to facilitate tissue repair, angiogenesis, and immune suppression (103–105).

**Table 3** summarizes the functional specializations of macrophage subsets, ranging from the microbicidal M1 phenotype to the varied M2a-d regulatory states, based on Roszer (106) and Duluc et al. (107) frameworks.

**Table 3:** Classification of macrophage polarization subsets.

Feature	M1 (classical)	M2a (alternative)	M2b (Type II)	M2c (acquired deactivation)	M2d* (TAM-like)
Inducing Stimuli	IFN-γ LPS, GM-CSF	IL-4, IL-13, Fungal/Helminth infection	Immune Complexes (ICs), IL-1R, TLR agonists	IL-10, TGF-β, Glucocorticoids (GCs)	IL-6, LIF, Adenosine
Marker Expression	CD86, CD80, CD68, MHC II,IL-1R, TLR2, TLR4, iNOS, SOCS3	CD163, MHC II, SR, CD206 (MMR), CD200R, TGM2, DecoyR, IL-1R II. *Murine:* Ym1/2, Fizz1, Arg-1	CD86, MHC II	CD163, TLR1, TLR8	VEGF
Cytokine Profile	TNF-α, IL-1β, IL-6, IL-12, IL-23	IL-10, TGF-β, IL-1ra	IL-10, TNF-α, IL-1, IL-6	IL-10, TGF-β	IL-10, IL-12, TNF-α, TGF-β
Chemokine Secretion	CXCL10, CXCL11, CCL5, CCL8, CCL9, CCL2, CCL3, CCL4	CCL17, CCL22, CCL24	CCL1	CCR2, CCL2	CCL5, CXCL10, CXCL16
Primary Biological Role	Microbicidal activity, Th1 priming, tumor suppression.	Wound healing, tissue repair, Th2 response, fibrosis.	Immunoregulation, Th2 promotion, humoral immunity.	Efferocytosis (clearing apoptotic cells), matrix remodeling.	Angiogenesis, tumor growth, and immunosuppression.

^*^The M2d subtype was proposed primarily by Duluc et al. (2007), based on leukemia inhibitory factor and IL-6 stimulation of monocytes, and represents a TAM-like state associated with angiogenesis and immune suppression. This classification is less widely validated than the M2a–M2c subtypes and remains an active area of investigation. Some authors instead classify this population within the broader M2 spectrum rather than as a discrete subtype.

### 4.2 Tumor-associated macrophages

**Figure 3:**
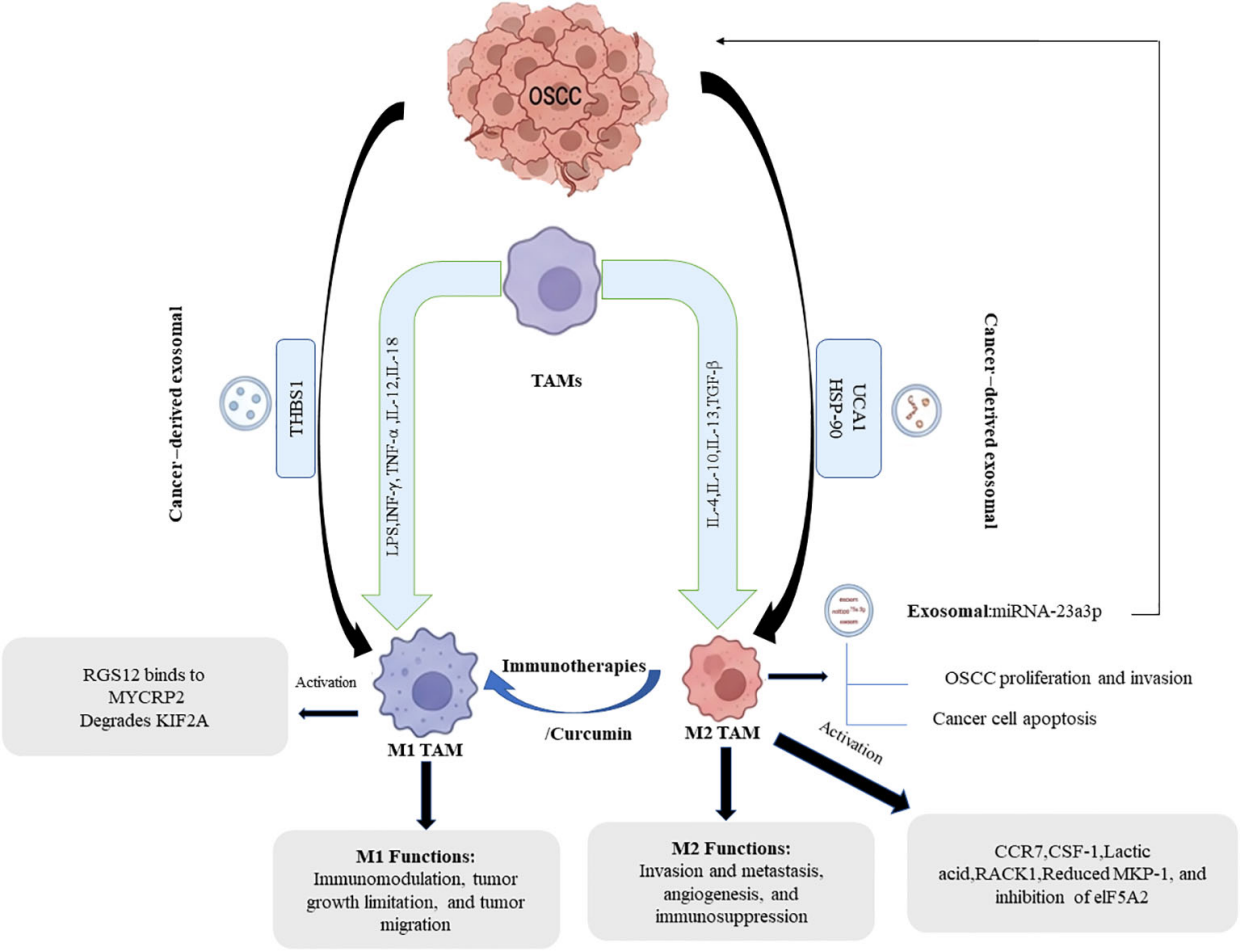
Molecular signaling pathways and exosomal crosstalk governing TAM polarization in the OSCC microenvironment. The phenotypic orientation of tumor-associated macrophages (TAMs) within oral squamous cell carcinoma (OSCC) is determined by a complex interplay of cytokines, intracellular proteins, and cancer-derived factors. Pro-inflammatory activation into the M1 phenotype is driven by LPS, IFN-γ, TNF-α, IL-12, and IL-18, a process further augmented by cancer-derived exosomal THBS1. Intracellularly, this M1 shift is mediated by RGS12 binding to MYCBP2, which triggers the degradation of KIF2A. Functionally, M1 TAMs serve to limit tumor growth and migration while promoting immunomodulation. Conversely, M2 polarization is induced by IL-4, IL-10, IL-13, and TGF-β, alongside cancer-derived exosomal UCA1 and HSP-90. The M2 state is maintained by various factors, including CCR7, CSF-1, RACK1, lactic acid, reduced MKP-1 levels, and the inhibition of eIF5A2. These M2 TAMs drive OSCC progression by fostering invasion, metastasis, angiogenesis, and immunosuppression. Furthermore, M2-derived exosomal miRNA-23a-3p creates a feedback loop that promotes OSCC cell proliferation and invasion while suppressing apoptosis. Therapeutic interventions, including immunotherapies and curcumin, aim to reprogram these pro-tumorigenic M2 TAMs back toward the antitumor M1 phenotype to inhibit OSCC progression.

TAMs are a key component of leukocytic infiltration in the tumor microenvironment (TME) and are frequently associated with poor prognosis in solid cancers (108). TAMs were thought to originate exclusively from circulating bone marrow-derived monocytes (BMDMs) recruited by chemokines such as CCL2 and CSF-1 (109). However, recent studies have indicated that specific TAMs (such as alveolar, brain, and liver macrophages) arise from prenatal embryonic progenitors (yolk sac or fetal liver) (110–113). TAMs exhibit high plasticity and are “educated” by the TME to adopt a phenotype that supports tumor survival. While early-stage tumors may transiently elicit an M1-like response, the established TME predominantly skews TAMs toward an M2-like, protumorigenic phenotype (114). This polarization is driven by tumor-derived lactic acid, hypoxia (via HIF-1α), and immunosuppressive cytokines (IL-10, TGF-β, and PGE2) (103, 115). Notably, TAMs often simultaneously express markers of both M1 and M2 phenotypes, suggesting a continuum rather than a fixed state (103, 114). TAMs facilitate cancer progression through three primary mechanisms: they secrete growth factors (EGF, VEGF, TGF-β, IL-6, Wnt ligands) that support tumor cell proliferation and angiogenesis; they produce matrix metalloproteinases that remodel the extracellular matrix (115). Furthermore, perivascular TAMs facilitate the intravasation of cancer cells into the bloodstream (116), and express immunosuppressive mediators (IL-10, TGF-β, PD-L1, arginase-1) that inhibit cytotoxic T-cell responses and foster an immune-privileged niche (117, 118). Chamseddine et al. summarized that high densities of M2-like TAMs correlate with advanced stage, metastasis, and poor prognosis across multiple solid tumors, and proposed targeting TAM recruitment, survival, or polarization as a promising strategy to enhance responses to chemo- and immunotherapy (119) (**Figure 3**).

## 5 Macrophage polarization in OSCC

Tumor-associated macrophages infiltrate OSCC tissues and represent a heterogeneous population influenced by tumor-derived cytokines and chemokines. In OSCC microenvironments, macrophages tend to adopt a phenotype that supports tumor growth and modulates local immune responses, although the precise molecular pathways remain incompletely characterized (120).

### 5.1 Predominance of M2 polarization in OSCC

In oral squamous cell carcinoma (OSCC), TAMs infiltrate tumor tissues and predominantly polarize toward an M2 phenotype, promoting progression through immunosuppression, angiogenesis, and enhanced cancer cell invasion. Origins include bone marrow-derived monocytes and tissue-resident (yolk sac-derived) macrophages (14, 23). Multiple studies demonstrate that M2-polarized macrophages are enriched in OSCC and are associated with malignancy progression. Wehrhan et al. showed that increased OSCC malignancy was accompanied by a shift from M1 to M2 macrophage polarization in regional lymph nodes, and that markers of aggressive tumor behavior were associated with enhanced M2 prevalence in the lymphatic stroma (121). A study by Kazumasa Mori et al. demonstrated that OSCC tissue specimens contain elevated CD163-positive M2 TAMs and that higher M2 macrophage density correlates with histopathological grade of OSCC malignancy (122). A systematic review by Alessandro Menna Alves et al. found that higher levels of pan-macrophage (CD68) and M2 macrophage (CD163) infiltration in OSCC are frequently associated with worse survival, reflecting the clinical significance of M2 polarization in prognosis (123). Beyond histological correlations, bioinformatic analysis of TCGA-HNSCC data has demonstrated that the ferroptosis suppressor FTH1 independently correlates with M2 macrophage infiltration, while the ferroptosis driver SOCS1 independently correlates with M1 macrophage infiltration, establishing a direct molecular link between ferroptosis gene expression and macrophage polarization status in the HNSCC immune microenvironment (124). Further corroborating this, *GPX4* gene expression — the master ferroptosis suppressor — was found to be positively correlated with M2 macrophage markers in HNSCC tissues (125), suggesting that ferroptosis-resistant tumors harbor a more immunosuppressive macrophage microenvironment. High CD206+ TAM density is associated with advanced stage, lymph node metastasis, and unfavorable clinical outcomes in OSCC (122, 123). All of the above findings lend support to the theory that an M2-skewed macrophage profile in OSCC tissues promotes immunosuppression, tumor growth, and worse clinical outcomes.

Supporting this heterogeneity at the molecular level, a study conducted by Hu et al. has shown that the ferroptosis suppressor *FTH1* independently correlates with M2 macrophage infiltration, while the ferroptosis driver SOCS1 independently correlates with M1 macrophage infiltration (124), and that *GPX4* expression positively correlates with M2 macrophage markers (VSIG4, MS4A4A) in a pan-cancer analysis that included HNSCC (125). At the single-cell level, integrated scRNA-seq and bulk RNA-seq analysis of HNSCC has revealed that M2 TAMs comprise transcriptionally heterogeneous subpopulations with distinct molecular signatures, and that high M2 TAM infiltration identified at single-cell resolution independently associates with advanced clinical stage, lymph node metastasis, and reduced overall survival (P<0.001) (126) — reinforcing that the M2 TAM compartment in HNSCC is not a uniform population but a spectrum of functionally distinct states whose full characterisation requires single-cell approaches. Importantly, single-cell transcriptomic analysis of 5,902 HNSCC cells has identified ferroptosis pathway enrichment across tumor cell subpopulations, with ACSL1, SLC39A14 (the zinc/iron transporter ZIP14), TFRC, and PRNP emerging as central ferroptosis-related genes distinguishing subpopulations with differential ferroptosis potential; a higher ferroptosis potential index (FPI) score derived from this single-cell data correlated with worse overall survival (127) — suggesting that ferroptosis susceptibility varies at the subpopulation level in HNSCC and that iron transporter expression (TFRC, SLC39A14) is a key determinant of this variability.

### 5.2 Mechanisms driving M2 polarization in OSCC

Bidirectional interactions between OSCC cells and macrophages establish feedback loops. Tumor-derived factors (exosomes, lncRNAs, metabolites such as lactic acid, and proteins including HMGB1 and ENO1) induce M2 polarization, while M2 TAMs secrete cytokines (IL-6, TGF-β, IL-10) that enhance OSCC proliferation, invasion, and migration via STAT3, NF-κB, and ERK signaling pathways, Tumor-associated macrophages promote tumor growth or metastasis, tissue remodeling, and immunosuppression by producing mediators such as IL-6 and MSF to promote tumor invasion, and by promoting irregular skewing of dendritic cells and expansion of regulatory T cells, thereby facilitating tumor immune escape (23).

### 5.3 Microenvironmental and microbial regulation of TAM polarization

Macrophage polarization in OSCC is not solely tumor intrinsic but is influenced by surrounding conditions. Soluble factors produced by OSCC cells have been shown to promote polarization of macrophages toward protumoral phenotypes, including upregulation of typical M2 markers and cytokines, although detailed regulatory networks in OSCC remain under active investigation (128). Chronic inflammatory states, including periodontitis, have been linked to enhanced M2 polarization in OSCC tissues. Periodontitis-associated microbiota promotes M2 polarization, accelerating OSCC progression, with higher M2 infiltration (24.97% ± 4.41% vs. 5.75% ± 0.52% in non-periodontitis models) and upregulated Arg1, IL-10, and CD206 (129). In OSCC, dysbiosis of the oral microbiota skews macrophages toward an immunosuppressive M2 phenotype. M2-like TAMs then contribute to tumor progression by secreting anti-inflammatory cytokines (IL-10, TGF-β), promoting angiogenesis, and expressing immune checkpoint ligands such as PD-L1 (15). Multiple bidirectional signaling loops between OSCC cells and TAMs have been identified that promote invasion and metastasis via STAT3 and NF-κB activation. These include: the Fusobacterium nucleatum–CXCL2 axis promoting macrophage recruitment; the THBS1/IL-6 feedback loop amplifying STAT3 activity; the PAI-1/IL-8 interaction supporting invasion; lactic acid signaling through GPNMB that drives M2 polarization; and the HMGB1/IL-6 axis activating NF-κB in macrophages (23). Decreased levels of C1QBP in OSCC cells enhanced macrophage polarization toward the M2 phenotype and facilitated tumor progression by activating pivotal elements of the tumor necrosis factor (TNF) signaling pathway, notably TRAF2 and CCL2 (130). In OSCC, dysbiosis of the oral microbiota, characterized by an overrepresentation of species such as Fusobacterium *nucleatum* and *Porphyromonas gingivalis*, works as a chronic inflammatory trigger, promoting epithelial-mesenchymal transition (EMT), immune evasion, and tumor formation. These pathogenic bacteria activate innate immune signaling pathways, such as TLRs and CSF-1R, thereby inducing macrophages to adopt an immunosuppressive M2 phenotype (15). Fusobacterium nucleatum (Fn) promotes M2 polarization, macrophage recruitment, and tumor cell proliferation, all of which aid in the advancement of OSCC (131).

### 5.4 M1 macrophages: context and complexity

While M2 polarization is generally associated with tumor-promoting functions, the role of classically activated or M1 macrophages in OSCC is complex and may vary by context and signaling environment: M1 macrophages are traditionally characterized as pro-inflammatory and anti-tumoral; however, in some cancer models, M1-like macrophage responses can paradoxically contribute to proliferation or invasion through specific cytokine-mediated pathways. Chunxu Lv et al. found that M1 macrophages promote survival, invasion, proliferation, migration, and xenograft formation in OSCC cells. M1 macrophages’ protumor impact is partially mediated by GDF15-induced ErbB2 phosphorylation (132). M1-like TAMs may cause a mesenchymal/stem-like phenotype of oral squamous cell carcinoma (OSCC) through the IL6/Stat3/THBS1 feedback loop. These groundbreaking findings revealed M1-like TAM-regulated mechanisms as potentially tumor-promoting in the setting of the OSCC immunomicroenvironment (133).

## 6 Molecular signaling in TAM polarization

**Table 4** summarizes major signaling axes identified in experimental and clinical studies that contribute to macrophage skewing toward a protumoral (M2) phenotype within the OSCC tumor microenvironment, including the IL-10/STAT3 axis, NF-κB signaling, Axl/PI3K/Akt/NF-κB pathways, chemokine-mediated feedback loops, and IL-6/STAT3 reinforcement mechanisms. Each pathway has been linked to immunosuppressive macrophage functions and OSCC progression in peer-reviewed evidence.

**Table 4:** Molecular pathways governing TAM polarization in OSCC.

Molecular pathway	Role in macrophage polarization	Reference
IL-10/STAT3 Signaling	Promotes M2 polarization and immunosuppressive phenotype	Faustino J Suárez-Sánchez et al. reported that IL-8 from tumor cells stimulates CD163^+^ M2 macrophages to produce IL-10, which activates STAT3 and induces PD-L1 overexpression, thereby linking the STAT3 pathway to the M2 phenotype and immune escape (135).
NF-κB Pathway	Tumor-derived factors activate NF-κB in macrophages, leading to M2 skewing and pro-tumoral mediator expression	OSCC cell factors induce NF-κB activation in macrophages; NF-κB promotes bidirectional crosstalk and increased aggressiveness (120).
Axl/PI3K/Akt/NF-κB Axis	OSCC cells use Axl signaling to promote M2 phenotype via a cascade involving PI3K/Akt and NF-κB	conditioned medium from OSCC cells activated Axl signaling, increasing CD206^+^ M2 TAMs; blocking Axl, PI3K/Akt, or NF-κB reduced M2 induction (136).
Chemokine-Mediated NF-κB Activation, such as CXCL2	Oral dysbiosis, such as *Fusobacterium nucleatum*, activates NF-κB into CXCL2, then recruits macrophages & M2 polarization	*Fusobacterium nucleatum* promotes OSCC via NF-κB to CXCL2, enhancing macrophage recruitment and polarization (131).
IL-6/STAT3 Feedback Loops	TAMs secrete IL-6, then STAT3 activation in OSCC cells/macrophages reinforces M2 state and tumor progression	OSCC cells and TAMs engage in feedback loops such as (IL-6, HSP27) that activate STAT3 and promote M2 functions, including invasion and chemoresistance (137).

A further oncogenic axis linking ferroptosis resistance to macrophage polarization in OSCC was identified by Qiao et al., who showed that ETS1 transcriptionally upregulates NXPH4, which suppresses GPX4 expression and macrophage M2 polarization simultaneously; silencing NXPH4 promoted ferroptosis markers (elevated MDA, ROS) and reduced M2 polarization in OSCC cell lines, identifying the ETS1/NXPH4 axis as a potential co-regulator of ferroptosis resistance and immunosuppressive macrophage programming (134).

## 7 Iron homeostasis–macrophage crosstalk

A critical and underappreciated dimension of the iron–macrophage axis is how iron metabolism status directly governs macrophage susceptibility to ferroptosis — and how that ferroptosis, in turn, reshapes the immunological tone of the tumor microenvironment. M1-polarized macrophages exhibit high intracellular iron sequestration through upregulation of ferritin H and suppression of FPN, yielding an elevated retained iron pool that sensitizes them to ferroptosis under oxidative stress (33, 138). By contrast, M2-like TAMs maintain higher ferroportin-mediated iron export and constitutively elevated GPX4 activity, conferring relative ferroptosis resistance that may permit their survival and immunosuppressive persistence within iron-rich tumor niches (125, 138). The immunological consequences of macrophage ferroptosis extend beyond simple cell loss: ferroptotic immune cells release damage-associated molecular patterns that activate innate immune sensing pathways and may remodel the TME from an immunologically cold toward a more T-cell-permissive state (139, 140). This positions macrophage-intrinsic ferroptosis as an upstream immunological event that potentiates the anti-tumor T-cell cascade; tumor cell ferroptosis — discussed in the following sections — then acts as a complementary downstream mechanism that further amplifies immunogenic signals and synergizes with ICI therapy.

### 7.1 Macrophages as central regulators of iron metabolism and immune function

Macrophages occupy a pivotal position at the interface between iron homeostasis and immune regulation. Beyond their canonical roles in innate immunity, macrophages are key regulators of systemic and local iron metabolism, owing to their capacity to sequester, store, recycle, and release iron (141). Importantly, macrophage polarization is closely coupled to the expression of iron-handling genes, indicating that immune activation states dictate distinct iron metabolic programs. In the tumor microenvironment, macrophage infiltration and polarization are frequently associated with divergent clinical outcomes, and TAMs often display iron metabolic phenotypes that differ markedly from those of macrophages in non-malignant tissues. These observations underscore the tight integration of iron metabolism with macrophage functional specialization in cancer (30).

### 7.2 Differential iron handling in macrophage polarization

Iron regulates macrophage polarization in various ways, including cellular signaling, metabolism, and epigenetic control (142). Polarized macrophages exhibit fundamentally distinct biological and metabolic properties, including divergent iron-handling strategies. Classically activated (M1) macrophages, induced by pro-inflammatory stimuli, adopt an iron-retentive phenotype characterized by iron sequestration via ferritin H upregulation and suppression of ferroportin-mediated export (138, 143). Iron acquisition in M1 macrophages occurs through transferrin receptor-1 (TfR1)–mediated uptake of transferrin-bound iron and through divalent metal transporters such as ZIP8 (144) and ZIP14 (145). In contrast, alternatively activated (M2) macrophages are specialized for iron recycling and export. In these cells, intracellular ferrous iron is oxidized and efficiently exported via the sole known cellular iron exporter, ferroportin (FPN). This polarization-dependent divergence in iron handling reflects the distinct functional roles of M1 and M2 macrophages in host defense, tissue repair, and tumor biology (138, 146). Upon alternative activation, macrophages upregulate ferroportin, enabling efficient iron release and thereby contributing to local iron provision for neighboring parenchymal and stromal cells. This ferroportin-high, iron-exporting state contrasts with inflammatory contexts in which hepcidin-driven ferroportin downregulation leads to iron retention within macrophages and supports a more pro-inflammatory program (143, 147–149). Macrophage ferritin, composed of H- and L-chain subunits, serves as a critical intracellular iron buffer that limits redox-active iron and constrains iron-dependent oxidative stress. Polarization-specific regulation of ferritin shapes iron availability: M1 macrophages generally display higher ferritin H expression and robust iron sequestration, whereas certain M2 populations exhibit comparatively reduced ferritin-mediated storage and a more readily mobilizable iron pool. Iron availability can influence the metabolic reprogramming of macrophages, thereby regulating their polarization state. Studies show that iron accumulation inhibits the STAT6 activation - via hepcidin upregulation-, promoting pro-inflammatory macrophage polarization (M1-like) (142). At the same time, iron overload can induce ferritin upregulation, and some studies indicate that excessive ferritin may dampen STAT6 activation and thereby restrain M2 polarization under conditions of chronic iron excess (150, 151). Iron catalyzes the generation of reactive ROS via Fenton chemistry, and changes in ROS levels constitute a key mechanistic link between iron availability and macrophage polarization. While multiple reports connect iron-induced ROS to pro-inflammatory, M1-like programs, low to moderate oxidative cues can also reprogram metabolism toward oxidative phosphorylation and favor anti-inflammatory gene expression patterns typical of M2 macrophages in specific tissue contexts (152). For example, in wound repair, iron-enriched environments skew macrophages towards an M2-like, pro-resolving phenotype, in part through ROS-dependent modulation of mitochondrial metabolism and redox-sensitive transcriptional regulators (153).

### 7.3 Iron status modulates macrophage polarization phenotypes

Iron availability itself acts as a regulatory signal that shapes macrophage polarization and immune responsiveness. Iron is indispensable for cellular metabolism, yet perturbations in iron homeostasis can profoundly influence innate and adaptive immune functions (154). In macrophages, changes in intracellular iron levels modulate inflammatory signaling pathways and polarization outcomes. Experimental studies demonstrate that iron loading attenuates the expression of M1-associated co-stimulatory molecules, such as CD86 and MHC class II, while simultaneously suppressing lipopolysaccharide-induced hepcidin expression. Elevated intracellular iron has been associated with a shift toward M2-like phenotypes and a dampening of pro-inflammatory responses. These findings indicate that iron status is not merely a metabolic parameter but an active determinant of macrophage functional identity (33).

### 7.4 Tumor cell–imposed iron restriction and polarization of TAM

Emerging evidence suggests that tumor cells can actively reshape macrophage polarization by manipulating iron availability within the tumor microenvironment. In cancer models, especially in hepatocellular carcinoma (HCC), M2-like TAMs exhibit lower intracellular ferrous iron levels compared with M1-like macrophages. Mechanistically, tumor cells compete with macrophages for iron through transferrin receptor–mediated uptake, thereby inducing iron deprivation within macrophages. This iron restriction stabilizes hypoxia-inducible factor-1α (HIF-1α) and promotes the transcription of M2-associated genes, ultimately driving immunosuppressive TAM polarization (35). These observations reveal iron competition as a previously underappreciated mechanism by which tumors modulate macrophage phenotype and immune escape.

### 7.5 Iron metabolism in TAM and prognostic implications

Dysregulated iron metabolism is increasingly recognized as a hallmark of cancer, with important implications for macrophage function and disease progression. Within the tumor microenvironment, TAMs commonly adopt an iron-release phenotype that facilitates iron availability to malignant cells, thereby supporting tumor growth and survival. In addition to iron delivery, TAMs secrete pro-angiogenic and tissue-remodeling factors that further promote tumor progression (29, 155).

Collectively, these findings highlight iron metabolism as a critical determinant of macrophage polarization and function in cancer. The interplay between iron handling and TAM biology provides a mechanistic link between metabolic dysregulation and immune modulation, with potential relevance for prognosis and therapeutic targeting. These pathways are highly relevant to OSCC, where hypoxia, oxidative stress, and M2-dominant TAMs coexist.

### 7.6 Iron metabolism, ferroptosis susceptibility, and macrophage immune function

The relationship between iron metabolism and macrophage polarization extends beyond signaling pathways to encompass differential ferroptosis susceptibility across macrophage phenotypes. M2-polarized TAMs, which adopt an iron-exporting phenotype to support tissue remodeling, concurrently upregulate ferroptosis-suppressive effectors, including Nrf2-driven transcription of GPX4 and SLC7A11 (125, 142). This coordinated upregulation of antioxidant defenses renders M2-like TAMs comparatively resistant to ferroptotic stimuli, while M1-like macrophages — which sequester iron and produce higher intracellular ROS — may be more susceptible to iron-dependent lipid peroxidation (138, 142, 147). This bidirectional relationship has a direct immunological consequence: iron-loaded, ferroptosis-resistant M2 TAMs persist within the OSCC TME and continue suppressing CD8+ T-cell responses, whereas selectively inducing ferroptosis within the M2 TAM compartment has been demonstrated using mannose-functionalized porous silicon nanoparticles loaded with erastin (Man@pSiNPs-erastin), which target CD206^+^ M2-like TAMs via the mannose-xCT axis — simultaneously depleting immunosuppressive cells, releasing pro-inflammatory signals, and enhancing the efficacy of anti-PD-L1 immunotherapy in preclinical HCC models (156, 157) — could simultaneously deplete immunosuppressive cells and release pro-inflammatory signals that reshape the immune microenvironment. While direct evidence for this mechanism in OSCC is currently absent and must be confirmed experimentally, the convergence of iron-handling biology and ferroptosis regulation within the macrophage compartment represents a conceptually important and underexplored dimension of the iron–TAM–ICI resistance axis.

### 7.7 TAM-derived exosomes as mediators of ferroptosis suppression in HNSCC

An important but underappreciated mechanism by which M2-polarized TAMs sustain ferroptosis resistance in head and neck squamous cell carcinoma is exosome-mediated intercellular communication. In laryngeal squamous cell carcinoma — a closely related head and neck malignancy — M2 macrophage-derived exosomes were shown to restore GPX4 expression and glutathione levels in erastin-treated tumor cells, reversing ferroptotic cell death and partially rescuing tumor cell proliferation (158). This exosomal ferroptosis inhibition operates through a distinct molecular axis: tumor-associated macrophage-derived exosomes enriched in annexin A3 (ANXA3) were demonstrated to inhibit ferroptosis in laryngeal cancer cells through an ATF2-CHAC1 signaling axis, in which ANXA3 stabilized the transcription factor ATF2, which in turn regulated CHAC1 expression to suppress lipid peroxide accumulation; this process was mechanistically associated with lymphatic metastasis (159). Taken together, these findings from HNSCC models reveal that M2 TAMs do not merely create a permissive immune environment for tumor growth — they actively transmit ferroptosis-suppressive signals to tumor cells via exosomal cargo, thereby protecting malignant cells from iron-dependent cell death. Whether analogous TAM-derived exosomal mechanisms operate in OSCC specifically remains an important open question, warranting direct experimental investigation.

The bidirectional relationship between TAM polarization and ferroptosis is summarised in **Table 5**, which integrates experimental, nanomedicine, and bioinformatic evidence from HNSCC and OSCC models.

**Table 5:** TAM–ferroptosis crosstalk in head and neck/oral squamous cell carcinoma: evidence from experimental and bioinformatic studies.

TAM source/interaction	Molecular mechanism	Effect on ferroptosis in tumor/TAM	Cancer model	Reference
A. M2 tumor-associated macrophages suppress ferroptosis in tumor cells				
M2-TAM conditioned medium → OSCC cells	M2 macrophage secretome upregulates GPX4 protein and restores GSH levels in tumor cells; reverses erastin-induced lipid peroxidation markers (ROS↓, MDA↓)	M2-CM suppresses ferroptosis in OSCC cells; restores proliferation, migration, and invasion capacity. CD206^+^ expression in tissue correlates positively with GPX4 protein (r>0, p<0.05)	*In vitro* + human OSCC tissue (IHC)	Su et al., 2026 (160)
M2 macrophage-derived exosomes (M2-exos)	M2-exos transferred to erastin-treated LSCC cells restore GPX4 mRNA and protein expression; restore GSH; reduce ROS and MDA levels	Exosomal transfer from M2 TAMs inhibits ferroptosis in HNSCC tumor cells; partially reverses erastin-induced cell death (p<0.05 for all ferroptosis markers)	*In vitro*, LSCC TU212 cells + IL-4-polarised M2 macrophages	Xu LC et al., 2022 (158)
TAM-derived ANXA3-rich exosomes	ANXA3 in exosomes inhibits ubiquitination of ATF2; ATF2 (transcription factor) drives CHAC1 expression → suppresses lipid peroxide accumulation and ferroptotic cascade	TAM exosomes inhibit ferroptosis via ATF2-CHAC1 axis; promotes lymphatic metastasis in LSCC (p<0.01). ANXA3 simultaneously drives M2 polarisation via AKT-GSK3β-β-catenin	Human LSCC tissues (proteomics) + *in vitro*/*in vivo*	Xu L et al., 2024 (159)
IGF2BP2 (RNA-binding protein) – NRF2–SLC7A11/GPX4 axis; M1 exosomes as therapeutic vector	IGF2BP2 stabilises NRF2 mRNA post-transcriptionally; sustains NRF2–SLC7A11/GPX4 antioxidant axis; M1 macrophage-derived exosomes fused to liposomes (si@PLE) deliver IGF2BP2 siRNA to disrupt this axis	IGF2BP2 promotes ferroptosis resistance in HNSCC; M1 exosome nanocarrier suppresses IGF2BP2-NRF2 axis → sensitises tumor to ferroptosis; combined photothermal therapy suppresses tumour growth *in vivo*	*In vitro* + *in vivo* HNSCC models	Li M et al., 2026 (161)
B. Ferroptosis Induction Depletes M2 TAMs and Remodels the Immune Microenvironment				
RSL3-induced ferroptosis → TAM depletion	RSL3 (GPX4 inhibitor) induces ferroptosis in HNSCC xenograft; ferroptotic DAMPs (calreticulin, HMGB1) stimulate immunological remodelling; GPX4 negatively correlated with calreticulin in HNSCC tissue (IHC)	Ferroptosis reduces M2 TAMs and MDSCs; increases CD4^+^ and CD8^+^ T cells in TME. High GPX4 = poor OS in HNSCC tissue microarray	*In vivo* C3H/He HNSCC xenograft + human tissue microarray	Zhao et al., 2023 (162)
Cucurbitacin B (CuB) → ferroptosis in M2 macrophages	CuB induces ferroptosis specifically in M2 macrophages: Fe²^+^ accumulation, GPX4↓, SLC7A11↓, COX-2↑, MDA/ROS/LPO products released → reprograms M2→M1 phenotype via oxidative signalling	CuB induces ferroptosis in M2 TAMs → M2→M1 repolarisation; inhibits OLK→OSCC malignant progression *in vivo* (4NQO murine model); CuB-activated M2 ferroptosis inhibits DOK dysplastic cell proliferation	*In vivo* murine OSCC (4NQO) + *in vitro* co-culture (OLK/OSCC)	Cheng et al., 2025 (163)
Mn²^+^ nanoreactors (hMnL) → ferroptosis + STING activation	hMnL releases Mn²^+^ in acidic TME → STING pathway activation → DC maturation + M1 macrophage polarisation; simultaneously induces ferroptosis and ICD via ROS cascade. Transcriptome: Fth1, Hmox1, Calr differentially expressed	hMnL induces ferroptosis AND drives M1 polarisation; increases CD8^+^ T cell infiltration, reduces Tregs; superior tumour suppression *in vivo*; long-term biosafety confirmed	*In vitro* + *in vivo* HNSCC models	Li W et al., 2026 (164)
Iron-tannic acid MPN nanocomplex (MPN@Pg-OMVMel) via microneedle patch	MPN coating consumes intratumoral H_2_O_2_ → Fenton reaction → ferroptosis; photothermal effect (808 nm) induces ICD (calreticulin exposure, HMGB1 release); reprograms M2→M1 TAMs; enhances DC maturation	Fenton-ferroptosis + photothermal ICD reprograms M2→M1 TAMs in murine OSCC; superior tumour suppression and significantly reduced recurrence; robust antitumour immune response established	Murine OSCC model (*in vivo*)	Li A et al., 2026 (165)
C. Bioinformatic Evidence: Ferroptosis Gene Expression Correlates with Macrophage Infiltration in HNSCC				
SOCS1 (ferroptosis driver)/FTH1 (ferroptosis suppressor) — TCGA-HNSCC	SOCS1: ferroptosis driver; FTH1: ferroptosis suppressor. TIMER/GEPIA2/TIMER2 analysis of TCGA-HNSCC: correlation with immune infiltration. Both are independent prognostic factors by multivariate Cox	SOCS1 independently correlates with M1 macrophage infiltration; FTH1 independently correlates with M2 macrophage infiltration in HNSCC (TCGA). Both are independent prognostic factors	TCGA-HNSCC bioinformatic analysis (TIMER, GEPIA2, TIMER2)	Hu et al., 2021 (124)
GPX4 protein expression and M2 macrophage markers — HNSCC/ESCA pan-analysis	TIMER/GEPIA/Oncomine analysis: GPX4 expression positively correlated with monocyte markers (CD14, CD115) and M2 macrophage markers (VSIG4, MS4A4A) in HNSCC and ESCA	GPX4 expression positively correlated with M2 macrophage markers in HNSCC tissue (VSIG4, MS4A4A, CD14, CD115); ferroptosis-resistant tumours have more immunosuppressive macrophage microenvironment	Pan-cancer bioinformatic analysis (TIMER, GEPIA, Oncomine)	Shi et al., 2021 (125)
M2 macrophage–ferroptosis gene interaction score (MFRS) — TCGA-HNSCC + GEO, machine learning	CIBERSORT: low M2 infiltration = better prognosis. WGCNA + Spearman: 1551 M2-related genes; 40 overlap with ferroptosis genes → 11-gene MFRS model (LASSO + plsRcox; C-index 0.645). PRKCA as hub gene	Low MFRS = longer survival, more active TME, greater ICI sensitivity. High MFRS = better chemotherapy response. Directly links M2 macrophage and ferroptosis gene co-expression to clinical outcomes in HNSCC	TCGA-HNSCC + GEO bioinformatic analysis; 101 machine learning algorithms	Huang et al., 2025 (166)
CLDND1 — ferroptosis-resistant, immune-cold phenotype — OSCC (TCGA + functional validation)	CLDND1 overexpressed in OSCC; positively associated with NFE2L2, SLC7A11, SLC3A2, GPX4 (ferroptosis-resistant axis); CIBERSORT: high CLDND1 = macrophage/resting compartment enrichment, cytotoxic T-cell signal reduction. *In vitro*: CLDND1 knockdown ↓GPX4, ↑ferroptosis susceptibility (RSL3)	High CLDND1 = immune-cold, ferroptosis-resistant OSCC phenotype; enriched macrophage resting compartment; independently predicts poor OS (HR 1.51) and DSS (HR 1.61); CLDND1 knockdown sensitises to RSL3-induced ferroptosis	TCGA-HNSCC/OSCC + functional validation (WSU-HN30, CAL27)	Zang et al., 2025 (167)
ETS1/NXPH4 axis — OSCC macrophage M2 polarisation and GPX4 suppression	ETS1 transcriptionally activates NXPH4; NXPH4 silencing: ↑MDA, ↑ROS, ↓GPX4 expression, ↓M2 polarisation in OSCC cell lines. TIMER2.0 + flow cytometry confirm NXPH4–M2 correlation	ETS1/NXPH4 axis co-regulates GPX4-mediated ferroptosis resistance AND M2 macrophage polarisation in OSCC; identifies novel molecular link between ferroptosis suppression and immunosuppressive macrophage programming	*In vitro* OSCC (CAL-27, SCC-25) + *in vivo* xenograft + TIMER2.0	Qiao et al., 2026 (134)

TAM, tumor-associated macrophage; OSCC, oral squamous cell carcinoma; HNSCC, head and neck squamous cell carcinoma; LSCC, laryngeal squamous cell carcinoma; GPX4, glutathione peroxidase 4; SLC7A11, solute carrier family 7 member 11; GSH, glutathione; ROS, reactive oxygen species; MDA, malondialdehyde; LPO, lipid peroxidation; IHC, immunohistochemistry; ICD, immunogenic cell death; DAMP, damage-associated molecular pattern; HMGB1, high-mobility group box 1; TME, tumor microenvironment; MDSC, myeloid-derived suppressor cell; M2-CM, M2-conditioned medium; OLK, oral leukoplakia; DOK, dysplastic oral keratinocyte; DC, dendritic cell; OS, overall survival; DSS, disease-specific survival; TCGA, The Cancer Genome Atlas; TIMER, Tumor Immune Estimation Resource; WGCNA, weighted gene co-expression network analysis; MFRS, macrophage-ferroptosis risk score; CIBERSORT, computational algorithm for immune cell deconvolution; LASSO, least absolute shrinkage and selection operator; STING, stimulator of interferon genes; IRF1, interferon regulatory factor 1; ANXA3, annexin A3; CHAC1, ChaC glutathione-specific gamma-glutamylcyclotransferase 1; ATF2, activating transcription factor 2; IGF2BP2, insulin-like growth factor 2 mRNA-binding protein 2; NRF2/NFE2L2, nuclear factor erythroid 2-related factor 2; NXPH4, neurexophilin-4; ETS1, E26 oncogene homolog 1; CLDND1, claudin domain containing 1; FTH1, ferritin heavy chain 1; SOCS1, suppressor of cytokine signaling 1; PRKCA, protein kinase C alpha.

## 8 Iron homeostasis, macrophage polarization, and resistance to immune checkpoint inhibitors in OSCC

Having established how iron loading drives M2 macrophage polarization through HIF-1α/IL-10/STAT6 signaling, and how ferroptosis operates as an iron-dependent cell death pathway in OSCC tumor cells in the previous Sections, this section examines how these two axes converge to determine resistance to immune checkpoint inhibitors’.

### 8.1 Clinical landscape of immune checkpoint inhibitors in OSCC and HNSCC

Immune checkpoint inhibitors (ICIs) targeting the PD-1/PD-L1 axis have transformed the management of recurrent and metastatic head and neck squamous cell carcinoma (HNSCC), a category encompassing OSCC. The landmark Phase III CheckMate-141 trial demonstrated that nivolumab, an anti-PD-1 monoclonal antibody, significantly improved overall survival compared to investigator’s choice single-agent chemotherapy (median OS 7.5 vs 5.1 months; HR 0.70; 97.73% CI, 0.51–0.96) in platinum-refractory recurrent/metastatic HNSCC, leading to its regulatory approval (19). In parallel, the KEYNOTE-048 trial established pembrolizumab — alone or combined with platinum chemotherapy — as a first-line standard for recurrent/metastatic HNSCC, with durable benefit observed particularly in patients with a combined positive score (CPS) ≥1 or ≥20 (17, 18). Despite these advances, objective response rates with ICI monotherapy remain modest, typically ranging from 13–17%, and approximately 60% of patients derive no durable clinical benefit (20, 21). In the specific context of OSCC, which arises predominantly from the oral cavity rather than the oropharynx, response rates may be even lower, in part because oral cavity carcinomas are predominantly HPV-negative and display a more immunosuppressive TME than their oropharyngeal counterparts (168). These observations underscore the urgent need to understand the dominant resistance mechanisms operating within the OSCC tumor microenvironment (**Table 6**).

**Table 6:** Key immune checkpoint inhibitor clinical trials in recurrent/metastatic HNSCC (including OSCC) with relevance to the iron–macrophage–immunotherapy axis.

Trial	Phase	Agent(s)	Setting/line	ORR (%)	Key outcome	Reference
CheckMate-141	III	Nivolumab vs IC	R/M HNSCC, 2nd line post-platinum	13.3 vs 5.8	Improved OS (7.5 vs 5.1 mo; HR 0.70). First ICI approval in HNSCC.	(19)
KEYNOTE-048	III	Pembrolizumab ± chemo vs EXTREME	R/M HNSCC, 1st line	19.1 (pembro mono, CPS≥1)	OS benefit for pembro mono (CPS≥1: 12.3 vs 10.3 mo) and pembro+chemo (13.0 vs 10.7 mo) vs EXTREME.	(17, 18)
KEYNOTE-012	Ib	Pembrolizumab	R/M HNSCC, PD-L1+ ≥1%	18	Early signal establishing PD-1 activity in HNSCC; responses in HPV+ (25%) and HPV− (14%) patients.	(187)
KEYNOTE-055	II	Pembrolizumab	R/M HNSCC, post-platinum + cetuximab	16	Confirmed activity of pembrolizumab in heavily pre-treated HNSCC; 12-mo OS rate 38%.	(188)
EAGLE (Durvalumab)	III	Durvalumab ± tremelimumab vs SoC	R/M HNSCC, 2nd line	17.9 vs 17.6 vs 12.0	Neither arm improved OS vs SoC; the combination ICI did not add benefit over monotherapy in the unselected population.	(189)

R/M, recurrent/metastatic; HNSCC, head and neck squamous cell carcinoma; IC, investigator’s choice; CPS, combined positivity score; ORR, objective response rate; OS, overall survival; SoC, standard of care; pembro, pembrolizumab; chemo, platinum + 5-FU.

### 8.2 TAM-mediated immunosuppression as a driver of ICI resistance in OSCC

M2-polarized tumor-associated macrophages represent one of the most mechanistically compelling drivers of ICI resistance in OSCC. The fundamental mechanism involves a multilayered suppression of T-cell-mediated anti-tumor immunity that operates both upstream and downstream of the PD-1/PD-L1 axis. First, M2-like TAMs express high levels of PD-L1 on their surface, creating an immunosuppressive TME that directly engages PD-1 on tumor-infiltrating CD8^+^ T cells and promotes T-cell exhaustion; an OSCC-specific meta-analysis confirmed that CD163^+^ M2-TAM infiltration is associated with poor prognosis and PD-L1 modulation in this cancer type (20, 47, 169). In OSCC, tumor-derived IL-8 appears to promote monocyte differentiation toward CD206+ TAMs (170), while separate OSCC studies show that TAM-associated IL-10 is linked to PD-L1 expression (171), and that OSCC-derived GM-CSF can drive PD-L1 upregulation through JAK2/STAT3 signaling (172). Second, the canonical immunosuppressive cytokines produced by M2-like TAMs — particularly IL-10 and TGF-β — suppress dendritic cell maturation and antigen presentation, impair cytotoxic T-lymphocyte (CTL) priming, and promote Treg expansion, collectively producing an immunologically ‘cold’ tumor microenvironment that is intrinsically resistant to PD-1 blockade (119). Third, M2-like TAMs secrete immunosuppressive mediators that impair antigen-presenting cell function by downregulating MHC class II expression through IL-10/STAT3-dependent mechanisms, thereby limiting effective CD4^+^ T-cell activation (173–176). In parallel, TAMs exhibit reduced capacity for antigen processing and cross-presentation, further constraining the generation of *de novo* tumor-specific CD8^+^ T-cell responses that would otherwise be amplified by ICI therapy (177, 178).

### 8.3 Iron loading of TAMs potentiates PD-L1 expression and ICI resistance

Emerging evidence indicates that iron metabolic status within TAMs directly modulates their immunosuppressive capacity and their expression of immune checkpoint ligands, establishing a mechanistic bridge between iron homeostasis dysregulation and ICI resistance. Within iron-laden tumor microenvironments, macrophages may polarize toward M2-like states through HIF-1α/IL-10/STAT6 signaling (142, 179), and iron-loaded macrophage subsets have been shown to upregulate PD-L1 on their surface (32). In preclinical cancer models, iron-loaded macrophage subsets (iTAMs) have been shown to more potently suppress T-cell responses compared to iron-low macrophage populations, with this immunosuppressive function mechanistically regulated through heme-induced degradation of the transcriptional repressor Bach1 and downstream activation of the endothelin receptor type B (Ednrb) signaling axis — myeloid-specific Ednrb deletion reduces tumor growth and restores MHC II expression on TAMs (32). Long-term iron overload additionally promotes an anti-inflammatory macrophage state characterized by suppression of pro-inflammatory mediators and a relative increase in IL-10 and TGF-β-driven immunosuppression (32, 155). These data are derived from non-oral cancer models (primarily murine sarcoma and hepatocellular carcinoma), and direct evidence for iron-loaded immunosuppressive TAMs in human OSCC tissue has not yet been reported. However, the biological conditions required for this mechanism to operate are present in OSCC: TfR1 is significantly overexpressed at the invasive tumor front (51, 57), hepcidin dysregulation creates conditions favoring iron retention — a mechanism directly demonstrated in HNSCC cell lines, where hepcidin-mediated ferroportin internalization rescues cells from iron depletion-induced growth arrest (37, 55), and ulcerating oral tumors generate local hemoglobin-rich environments that provide additional iron substrate for macrophage uptake. Taken together, these converging features suggest that iron-mediated TAM immunosuppression may operate in OSCC, though this remains a hypothesis requiring direct experimental validation.

Providing the first direct clinical evidence in OSCC, Su et al. demonstrated in human tissue specimens that CD206 (M2 macrophage marker) and GPX4 expression are positively correlated in OSCC samples, and showed experimentally that M2 macrophage-conditioned medium upregulates ferroptosis resistance in OSCC cells, enhancing their proliferation, migration, and invasion capacity (160). This OSCC-specific finding directly supports the hypothesis that M2 TAM-rich microenvironments confer ferroptosis resistance to tumor cells, establishing a mechanistic basis by which M2 TAM infiltration — driven by iron loading — sustains both tumor aggressiveness and ICI resistance.

Despite direct experimental evidence linking intramacrophage iron status to immunosuppressive function specifically within OSCC tissue is limited, the mechanistic framework presented here is supported by data from preclinical cancer models, macrophage biology studies, and iron metabolism research in other tumor types (32, 35, 155, 179), and is grounded in the well-characterized M2-dominant, iron-rich, and hypoxic features of the OSCC microenvironment (23, 51). We propose this iron–TAM–immunosuppression axis as a testable hypothesis warranting systematic investigation through spatial transcriptomics, ex vivo iron-macrophage co-culture experiments, and biomarker-stratified analysis of ICI-treated HNSCC cohorts.

### 8.4 Ferroptosis, anti-tumor immunity, and the ICI connection

Iron-dependent cell death via ferroptosis occupies a critical immunological interface that is directly relevant to ICI responses in OSCC. Ferroptosis is increasingly recognized as a form of immunogenic cell death: ferroptotic tumor cells release damage-associated molecular patterns (DAMPs) and lipid oxidation products that stimulate dendritic cell maturation and activate anti-tumor CD8^+^ T-cell responses, thereby creating an immunologically favorable context for ICI therapy (37, 38). Conversely, CD8^+^ T cells activated by immune checkpoint–based immunotherapy promote ferroptosis in tumor cells by secreting IFN-γ, which downregulates SLC7A11 and SLC3A2, the two subunits of the glutamate–cystine antiporter system Xc^-^, thereby impairing cystine uptake, depleting glutathione, and enhancing lipid peroxidation and ferroptotic cell death (180–183).

In OSCC, Tumors with higher ferroptosis potential — characterized by low GPX4/SLC7A11 and high ACSL4 expression — tend to show features consistent with immune activity (184, 185). Ferroptosis-related gene signatures have been linked to immune cell infiltration patterns in OSCC cohorts, with high-risk ferroptosis gene scores correlating with reduced CD8^+^ T-cell, NK-cell, and iDC infiltration (54, 102). Importantly, experimental induction of ferroptosis by RSL3 in a HNSCC xenograft model significantly reduced M2-like TAMs and MDSCs while increasing CD4^+^ and CD8^+^ T-cell infiltration (162), supporting the concept that ferroptosis-sensitive tumors may be more amenable to immunotherapy. However, ferroptosis induction can also trigger compensatory immunosuppressive IL-1β release, highlighting the importance of carefully designed combination approaches (186), suggesting that ferroptosis-sensitive OSCC tumors may be more responsive to ICI. Integrating ferroptosis-related gene signatures (FRG scores) with immunological profiling could therefore represent a rational approach to identifying OSCC patients most likely to benefit from ICI therapy.

An experimental study conducted by Zhao et al., which was conducted in a HNSCC xenograft model, directly demonstrated that induction of ferroptosis by RSL3 significantly reduced M2-like tumor-associated macrophages and myeloid-derived suppressor cells (MDSCs) in the tumor microenvironment, while simultaneously increasing tumor-infiltrating CD4^+^ and CD8^+^ T cells; calreticulin and HMGB1 were identified as candidate mediators of this immunogenic remodeling (162). Furthermore, a machine-learning analysis of TCGA-HNSCC multiomics data identified 40 genes with overlapping M2 macrophage-related and ferroptosis gene functions, constructing a macrophage-ferroptosis risk score (MFRS) in which patients with low MFRS presented longer survival and greater sensitivity to immunotherapy (166). These convergent findings reinforce the concept that ferroptosis induction in OSCC reshapes the macrophage compartment from immunosuppressive to pro-inflammatory, creating conditions favorable for ICI responsiveness.

### 8.5 HPV status, iron metabolism, and differential ICI responses in OSCC

HPV status is a critical biological variable that profoundly influences both the immune landscape of OSCC/HNSCC and the response to ICI therapy. HPV-positive tumors — predominantly arising in the oropharynx — display a characteristically ‘hot’ immune microenvironment with increased infiltration of CD8^+^ T cells, higher T-cell receptor diversity, elevated immune cytolytic activity, and a T-cell-inflamed gene expression profile (168). A pooled analysis of ICI clinical trials demonstrated that HPV-positive HNSCC patients derive greater overall survival benefit from PD-1/PD-L1 inhibitors compared to HPV-negative patients (OS HR = 0.71, p = 0.02; ORR 21.9% vs 14.1%, OR = 1.79, p = 0.01) (168). In contrast, HPV-negative oral cavity OSCC — which constitutes the predominant OSCC subtype globally, driven by tobacco, alcohol, and betel nut — is characterized by a more immunosuppressive TME with higher M2-TAM infiltration, greater Treg/CTL imbalance, and lower baseline CD8^+^ T-cell density (20, 21). The relationship between HPV status and iron metabolism in the OSCC TME has not been studied, but represents an important gap: HPV-positive tumors, with their more inflamed microenvironments, may generate a distinct iron utilization pattern compared to HPV-negative tumors with M2-dominant macrophage infiltration. Future studies investigating iron-related protein expression (TfR1, ferritin, ferroportin, HO-1) stratified by HPV status are necessary to determine whether iron-targeted therapeutic strategies should be tailored to HPV subtype.

## 9 Approaches to modulating the iron metabolism-macrophage axis: therapeutic interventions and clinical translational implications

### 9.1 Strategic interventions for the modulation of iron metabolism and their associated challenges

Targeted modulation of iron metabolism demonstrates significant therapeutic potential in OSCC, but its clinical translation requires a deep understanding of the mechanisms regulating iron homeostasis within the oral tumor microenvironment. Existing research indicates that interventions targeting macrophage and cancer cell iron concentrations may influence tumor progression by modulating immune polarization states and cancer cell viability.

### 9.2 Iron chelation therapy

While iron chelation may appear to contradict the ferroptosis-induction strategies described later in this section — since ferroptosis requires available Fe²^+^ for Fenton-driven lipid peroxidation — these approaches operate in biologically distinct contexts and address different therapeutic objectives: chelation targets the excess labile iron that sustains rapid tumor cell proliferation (55, 190) and may reduce iron-loading-driven M2 TAM polarization within the TME (33, 142), whereas ferroptosis inducers exploit the GPX4/system Xc^-^ vulnerability already present within iron-loaded OSCC cells and do not require exogenous iron supplementation to be effective; the specific immunological and molecular context determining which strategy is optimal — and whether a sequential chelation-then-induction regimen could be more effective than either approach alone — is addressed through the biomarker-guided stratification framework discussed in the Translational Challenges subsection below.

The iron chelator deferoxamine (DFO), the most clinically studied siderophore-based chelator, exerts antiproliferative effects in cancer cells by depleting the labile iron pool essential for ribonucleotide reductase activity, thereby suppressing DNA synthesis and cell cycle progression (191). In HNSCC specifically, iron depletion via the chelator ciclopirox olamine (CPX) significantly reduced viability and clonogenic survival in multiple HNSCC cell lines while sparing normal oral epithelial cells, with iron confirmed as the critical mediator through rescue experiments (190). Synergism between iron chelation and radiation therapy has been hypothesized based on the iron dependence of DNA repair mechanisms, though systematic preclinical validation specifically with DFO in HNSCC models remains limited and represents an important research gap. In THP-1-derived macrophages, chronic iron overload promotes M2-like polarization characterized by upregulation of arginase and CD163, while iron chelation with DFO reverses this M2 skewing and modulates macrophage iron-handling phenotype (192). However, systemic iron chelation therapy faces significant challenges in OSCC, including the risk of anemia due to nonspecific iron depletion, potential impairment of immune cell function, and other systemic side effects that limit clinical application (191, 193). Moreover, DFO’s short half-life and poor oral bioavailability necessitate continuous intravenous infusion, complicating outpatient management for OSCC patients.

### 9.3 Modulating iron transport

Ferroportin (*SLC40A1*), the sole identified mammalian iron exporter, represents a critical therapeutic node in OSCC. Studies in metastatic HNSCC cell lines (HN12, JHU-022) demonstrate that ferroportin overexpression induces cell cycle arrest, DNA damage, and cellular senescence by depleting labile iron pools, effects that are rescued by hepcidin-mediated ferroportin internalization (55). Hepcidin antagonists or ferroportin stabilizers have been developed to counteract excessive hepcidin-induced ferroportin degradation, thereby promoting iron efflux from cells such as macrophages and enterocytes. However, because ferroportin is widely expressed beyond tumor‐associated macrophages — including in intestinal enterocytes and hepatocytes — achieving selective modulation of tissue iron without compromising systemic iron homeostasis remains a major translational challenge (194–198).

### 9.4 Ferroptosis modulation

Unlike pulmonary fibrosis, where ferroptosis inhibition is protective, OSCC presents a dual paradigm. In established OSCC, inducing ferroptosis represents a promising therapeutic strategy to eliminate therapy-resistant tumor cells, particularly those with mesenchymal phenotypes that resist apoptosis. Ferroptosis inducers such as RSL3 (GPX4 inhibitor), erastin (system Xc^-^ inhibitor), and sulfasalazine demonstrate selective cytotoxicity against OSCC cells while sparing normal fibroblasts (53).

Conversely, in premalignant conditions (oral leukoplakia, erythroplakia, oral submucous fibrosis), ferroptosis inhibitors (ferrostatin-1, DFO) may preserve epithelial integrity and prevent malignant transformation by preventing lipid peroxidation-driven DNA damage and compensatory proliferation of mutated cells (191). In an OSCC-specific context, cucurbitacin B was recently shown to induce ferroptosis selectively in M2 macrophages — evidenced by Fe²^+^ accumulation, GPX4 and SLC7A11 downregulation, and increased MDA, ROS, and lipid peroxidation products — resulting in reprogramming of M2 macrophages toward an M1 phenotype; *in vivo*, this mechanism significantly inhibited the progression of oral leukoplakia to OSCC in a murine 4NQO carcinogenesis model (163). This study provides the first direct evidence that pharmacological ferroptosis induction in M2 macrophages — rather than tumor cells — can suppress malignant progression in the oral cavity, establishing macrophage ferroptosis as a distinct and therapeutically actionable target. This context-dependent approach necessitates precise patient stratification based on disease stage and molecular profiling.

### 9.5 Nrf2 pathway modulation

The Nrf2 agonist dimethyl fumarate (DMF) demonstrates context-dependent effects in OSCC. At lower concentrations (<25 μM), DMF activates Nrf2, upregulating HO-1 and MnSOD, reducing oxidative stress, and inhibiting epithelial-mesenchymal transition (EMT) in CAL27 cells and orthotopic tongue cancer models (199, 200). However, at higher concentrations (>25 μM), DMF inhibits Nrf2 nuclear translocation by downregulating DJ-1, leading to increased oxidative stress and cytotoxicity in KRAS-mutated cancer cells (200, 201). This biphasic effect highlights the need for careful dose optimization in OSCC, where Nrf2 is often constitutively activated as a survival mechanism. ROS-responsive nanoparticle delivery systems, analogous to those demonstrated in pulmonary fibrosis models, may enable tumor-specific Nrf2 modulation while minimizing systemic exposure (179).

### 9.6 Heme oxygenase-1 inhibition

HO-1 serves as the central enzyme in heme degradation, catalyzing the conversion of heme to carbon monoxide (CO), biliverdin, and free ferrous iron. In nasopharyngeal carcinoma (NPC), a head and neck cancer closely related to OSCC, high HO-1 expression in TAMs significantly correlates with CD163+ M2 macrophage infiltration and predicts poor prognosis (202). It should be noted that NPC and OSCC, while both classified as head and neck cancers, are biologically distinct malignancies with different etiologies (EBV-associated vs. tobacco/alcohol/HPV-driven), anatomical locations, and immune microenvironment compositions; therefore, HO-1 data from NPC should be interpreted as suggestive of a potential role in OSCC rather than directly applicable without additional OSCC-specific validation. HO-1 drives macrophage polarization toward the pro-tumorigenic M2 phenotype through CO-mediated activation of the Nrf2/STAT6 axis (203). Zinc protoporphyrin IX (ZnPP), an HO-1 inhibitor, suppresses M2 polarization and enhances antitumor immunity (203). However, further research is needed to elucidate HO-1’s specific regulatory mechanisms in OSCC, particularly regarding its dual role in protecting normal oral mucosa from carcinogen-induced oxidative stress versus promoting established tumor progression.

### 9.7 Novel therapeutic strategies and future directions

Focusing on the intersection between macrophage polarization reprogramming and iron metabolism intervention reveals a therapeutically significant but pharmacologically complex interplay. Iron has been shown to negatively regulate miR-29a expression (142); because miR-29a promotes M2 polarization via the SOCS-1/STAT6 pathway, this suggests that low intracellular iron favors a more pro-inflammatory macrophage state, while iron supplementation — by suppressing miR-29a — could theoretically promote M2 skewing. Paradoxically, this means that iron chelation would be expected to upregulate miR-29a, which might enhance rather than inhibit M2-like polarization through this particular axis, even if it reduces tumor cell iron availability. This bidirectional complexity illustrates a recurring challenge in the field: interventions targeting iron metabolism can produce opposing effects on macrophage polarization through different molecular pathways, and the net immunological outcome will depend on the specific iron-sensing mechanism predominant in each cellular context. Preclinical validation using OSCC-specific models is essential before any iron-modulating strategy is advanced to clinical translation.

### 9.8 Combination strategies

Evidence indicates that targeting shared iron homeostasis between tumor cells and TAMs may represent a more effective therapeutic strategy than single-agent approaches. In HNSCC, tumor cells exhibit a heightened dependence on intracellular iron to drive progression and survival, making them highly sensitive to iron depletion (55). Specifically, iron chelation has been shown to significantly reduce HNSCC cell viability and clonogenic capacity (190), while recent evidence suggests that deferoxamine (DFO) effectively modulates iron-mediator expression to exert anti-tumorigenic effects in head and neck squamous cell models (190). Moreover, emerging evidence suggests that modulation of iron-dependent pathways, particularly ferroptosis, can enhance cisplatin sensitivity and overcome chemoresistance in OSCC, supporting a potential role for iron-targeting strategies in combination therapy, although direct evidence for DFO–cisplatin synergy in HNSCC remains limited. The combination of ferroptosis inducers (erastin, RSL3) with cisplatin effectively overcomes chemoresistance in OSCC by restoring ferroptotic cell death pathways (53). Mechanistically, erastin and RSL3 target key ferroptosis regulators, including system Xc^-^ and GPX4, while the Nrf2/HO-1 antioxidant axis functions upstream as a critical mediator of ferroptosis resistance in OSCC (53, 92).Circ_0000140 promotes cisplatin resistance in OSCC by functioning as a molecular sponge for miR-527, thereby relieving miR-527-mediated repression of *SLC7A11*. Silencing circ_0000140 restores ferroptosis—evidenced by increased intracellular iron and ROS—and enhances cisplatin sensitivity, confirming the functional importance of the circ_0000140/miR-527/*SLC7A11* axis in chemoresistance (63). These findings underscore that ferroptosis suppression is a hallmark of cisplatin resistance, and its reactivation is a promising strategy for chemosensitization.

### 9.9 Advanced targeted delivery systems

To enhance therapeutic targeting and specificity while minimizing systemic toxicity, researchers are increasingly focusing on oral cavity-directed delivery systems. Iron oxide nanoparticles (IONPs) offer unique advantages for OSCC therapy by reprogramming the tumor microenvironment (TME). Specifically, these nanoparticles have been shown to re-educate tumor-associated macrophages (TAMs) from an immunosuppressive M2 phenotype to a pro-inflammatory M1 phenotype, a mechanism rigorously demonstrated in breast cancer models (204). While this immunomodulatory potential is broad, its application to oral malignancies is further supported by OSCC-specific studies. For instance, superparamagnetic iron oxide nanoparticles (SPIONs) have been found to induce selective mitochondrial toxicity and reactive oxygen species (ROS) production in oral tongue squamous cell carcinoma (OTSCC) cells, while sparing normal tissue, as conducted by Afrasiabi et al. (205). Consequently, the integration of IONP-driven macrophage reprogramming with site-specific cytotoxic effects presents a robust, multi-modal strategy for the targeted treatment of OSCC. This effect is mediated, at least in part, through iron-induced ROS generation (206) and activation of pro-inflammatory signaling pathways, including TLR4-dependent mechanisms, which together promote macrophage activation and antitumor immune responses (207). Preclinical studies using clinically approved nanoparticles such as ferumoxytol have demonstrated their ability to inhibit tumor growth by promoting M1 macrophage polarization and enhancing antitumor immune responses. While additional mechanisms, including innate immune receptor signaling, have been proposed, current evidence most consistently supports ROS-driven macrophage reprogramming as the dominant pathway (208). The degradation of iron oxide nanoparticles contributes to intracellular iron release, which can catalyze Fenton-type reactions, leading to increased production of ROS and subsequent alterations in cellular redox balance and iron metabolism (152, 209). Mannose-modified nanoparticles can actively target tumor-associated macrophages (TAMs) through mannose receptor (CD206) engagement, enabling selective delivery of therapeutic agents to the tumor microenvironment (156). This platform provides a potential strategy for delivering iron-modulating compounds, such as iron chelators or ferroptosis-related agents (156, 164, 210, 211).

Innovative biomimetic approaches include M2 exosome-liposome nanohybrids that leverage the homing capabilities of exosomes derived from M2 macrophages to target tumor sites. Extending this approach, a photothermal-ferroptosis nanoplatform combining an iron-tannic acid metal-phenolic network with Porphyromonas gingivalis outer membrane vesicles (MPN@Pg-OMVMel), delivered via dissolvable microneedle patches, successfully reprogrammed M2 TAMs to an M1 phenotype and induced immunogenic cell death in murine OSCC models, achieving superior tumor suppression and reduced recurrence (165). Complementing this, manganese dioxide nanoparticles activating both ferroptosis and the STING innate immune pathway demonstrated concurrent induction of M1 macrophage polarization, dendritic cell maturation, and enhanced CD8^+^ T cell infiltration in HNSCC models, further establishing the principle that ferroptosis-inducing nanoplatforms can simultaneously remodel the macrophage and T-cell compartments of the TME (164).

A critical translational consideration for ferroptosis-based therapy in OSCC is the need to selectively target tumor cells and pro-tumoral M2 macrophages while preserving beneficial iron-rich immune populations — particularly M1-like macrophages that mediate anti-tumor immunity. Emerging evidence reveals that macrophage polarisation state intrinsically determines ferroptosis susceptibility: iNOS-derived nitric oxide in activated M1 macrophages modulates susceptibility to ferroptosis by inhibiting 15-lipoxygenase-driven pro-ferroptotic lipid peroxidation, whereas M2 macrophages — which do not express iNOS — lack this protection and are comparatively more susceptible to ferroptotic stimuli (212). This polarisation-dependent ferroptosis susceptibility creates a biological selectivity window that therapeutic strategies can exploit: ferroptosis induction preferentially eliminates immunosuppressive M2 TAMs while M1 anti-tumor macrophages retain intrinsic resistance through the iNOS/NO axis. At the molecular level, OSCC cells additionally harbour constitutively active Nrf2 — driven by KEAP1 inactivation — which paradoxically creates a pharmacological vulnerability: Nrf2 inhibitors such as brusatol (81), and carnosic acid (94) selectively collapse the ferroptosis-suppressive axis in Nrf2-dependent tumour cells, while normal macrophages relying on inducible rather than constitutive Nrf2 activation retain greater antioxidant reserve. Furthermore, direct GPX4 inhibition with RSL3 demonstrates selective cytotoxicity against OSCC cells while sparing normal oral fibroblasts (53, 92), and tumour-targeted delivery platforms — including mannose-modified nanoparticles that preferentially bind CD206^+^ M2 TAMs (156) and pH-responsive carriers that activate selectively in the acidic OSCC microenvironment (164, 165),— can concentrate ferroptosis-inducing payloads within malignant and pro-tumoral cells while limiting exposure to beneficial immune populations. Together, these biological and engineering principles provide a preclinical framework for immune-sparing ferroptosis therapy in OSCC, though systematic validation in orthotopic immunocompetent OSCC models is required before clinical translation.

These advanced delivery systems offer potential solutions for improving oral cavity targeting while reducing systemic exposure, though their efficacy and safety in the unique microenvironment of OSCC require systematic evaluation through rigorous preclinical and clinical studies.

### 9.10 Translational challenges and biomarker-guided stratification

Although targeting iron metabolism to modulate macrophage polarization shows promise, translation to OSCC remains challenging due to the complex tumor microenvironment. In OSCC, tumor-associated macrophages (TAMs) are highly plastic and contribute to tumor progression, invasion, and immunosuppression (23). Given this complexity, single-pathway targeting is unlikely to be sufficient, as macrophage function is regulated by multiple metabolic and signaling cues within the TME (23). Therefore, effective strategies will likely require combinatorial approaches integrating iron modulation with immunotherapy (PD-1/PD-L1) and oncogenic pathways (EGFR, PI3K/AKT) (213).

Future research should focus on developing precise biomarker-guided patient stratification strategies. Ferroptosis-related gene (FRG) signatures derived from TCGA and GEO datasets reveal differential expression patterns between OSCC tumors and normal oral epithelium, with high-risk FRGs predicting poor survival. Future investigation using independent OSCC cohorts from TCGA-HNSC or GEO, correlating iron-metabolism gene expression with M2-macrophage infiltration signatures, is needed to provide disease-specific validation of this hypothesis. A ferroptosis score (FPscore) correlates with tumor purity, immune cell infiltration, and patient prognosis; patients with high FPscore show longer survival and enhanced responses to immunotherapy (53). Integrating iron metabolism biomarkers—such as serum ferritin, transferrin receptor 1 (*TfR1*) expression, *GPX4* and *SLC7A11* levels, and lipid peroxidation markers—could enable personalized therapeutic selection. Patients with high iron burden and M2-dominant TAM infiltration may benefit from iron chelation combined with immunotherapy, whereas those with high GPX4-expressing, ferroptosis-resistant tumors may require combination strategies for ferroptosis induction.

Continuous optimization of oral cavity-specific delivery technologies and thorough exploration of synergistic effects between iron metabolism regulation and existing OSCC therapeutics (cisplatin, cetuximab, pembrolizumab) aim to provide patients with more effective and safer treatment options. Building on emerging scRNA-seq evidence that ferroptosis pathway activity and iron transporter expression (TFRC, SLC39A14) vary across HNSCC subpopulations (127), and that M2 TAMs themselves comprise transcriptionally heterogeneous states (126), future studies leveraging spatial transcriptomics integrated with single-cell data from OSCC-specific cohorts could define which TAM subsets and tumor subpopulations are most vulnerable to iron-dependent ferroptosis.

## 10 Conclusion and perspectives

### 10.1 Summary of key findings

OSCC is a progressive and often fatal malignancy involving complex interactions across multiple levels in its pathogenesis, including epithelial transformation, immune microenvironment dysregulation, and abnormal extracellular matrix remodeling. This review systematically examines the pivotal role of macrophages in OSCC, particularly focusing on the significance of M1/M2 polarization phenotype switching and iron metabolism dysregulation in tumor progression. Existing research indicates that M2 macrophages promote cancer cell proliferation, invasion, and chemoresistance by secreting factors such as TGF-β, VEGF, and IL-10. Meanwhile, alterations in iron homeostasis in both tumor cells and tumor-associated macrophages (TAMs) are increasingly recognized as modulators of macrophage polarization through pathways such as HIF-1α/STAT6 and redox-sensitive signaling. However, the effects of iron on macrophage polarization remain context-dependent, varying according to iron availability, tumor type, and microenvironmental conditions. Overall, dysregulated iron metabolism may contribute to OSCC progression and therapeutic resistance, although direct mechanistic evidence in OSCC remains limited and is largely extrapolated from other cancer models (**Table 7**).

**Table 7:** Summary of key reviews on crosstalk between iron homeostasis and macrophage polarization, with emphasis on mechanistic pathways, disease contexts, and implications for tumor-associated macrophages in oral squamous cell carcinoma.

Reference	Main focus	Key concepts on iron–macrophage crosstalk	Relevance to OSCC
Xia et al.,(2021) (142)	Mechanistic review of iron regulation of macrophage polarization across diseases	Describes how intracellular and extracellular iron levels, iron transporters (TfR1, DMT1, ferroportin), and storage proteins (ferritin) shape M1 vs M2 polarization via ROS, NF-κB, MAPKs, HIF-1α, and metabolic rewiring between glycolysis and oxidative phosphorylation.	Provides the mechanistic backbone for linking iron handling to TAM phenotypes in OSCC, such as (iron-releasing M2-like TAMs vs iron-sequestering M1-like TAMs), and for discussing signaling pathways potentially operative in oral tumors.
Minlan Luo et al., (179)	Organ-specific (lung) review on iron and macrophage polarization in pulmonary fibrosis	Integrates iron accumulation in lung tissue with activation of a HIF-1α/IL-10/STAT6 axis in macrophages, driving M2 polarization, fibroblast activation, and matrix deposition; discusses iron chelators, ferroportin modulation, and nanomedicine as therapeutic strategies.	Offers a disease model (fibrotic lung) where iron-rich microenvironments drive M2-biased macrophages; this can be extrapolated to OSCC stroma, where chronic inflammation and bleeding may generate iron-loaded, profibrotic, and pro-tumorigenic TAMs and suggest iron-targeted therapies.
Nathan C Winn et al., (2020) (147)	Tissue-resident macrophages as local regulators of iron (ferrostats)	Explains how macrophages orchestrate local iron flux through coordinated expression of uptake (TfR1, CD163), storage (ferritin), and export (ferroportin) systems, and how these iron circuits integrate with macrophage activation and tissue-specific immune responses.	Supports a conceptual framework in which OSCC-associated macrophages function as oral ferrostats, controlling iron availability to tumor cells and stromal elements; suggests evaluating ferroportin, ferritin, and CD163 as part of an “iron signature” of OSCC TAMs.
Recalcati S et al., (2019) (150)	Iron in macrophage immunometabolism	Details how iron availability influences macrophage metabolic programs (glycolysis, TCA cycle, oxidative phosphorylation) and how these metabolic states align with distinct polarization phenotypes and effector functions.	Provides rationale to integrate iron metabolism with OSCC TAM immunometabolism, such as (coupling iron status, mitochondrial function, and lactate production), and to discuss how metabolic interventions might co-target iron and polarization in OSCC.
Mertens C et al., (2021) (214)	Macrophage iron-related gene signatures across tissues and diseases	Synthesizes data on macrophage iron signatures (expression of H-/L-ferritin, ferroportin, hepcidin, heme-handling receptors) and shows how these signatures reflect and shape activation states (inflammatory vs reparative) in multiple pathologies.	Suggests that an OSCC study could define an oral TAM iron signature and correlate ferroportin/ferritin/heme-scavenger expression with M1/M2 markers, prognosis, and response to therapy.
Shuo Ni et al., (2022) (154)	Broad overview of iron in innate and adaptive immune regulation	Reviews how systemic and cellular iron metabolism modulate immune cell development, activation, and tolerance, including a section on iron-dependent regulation of macrophage polarization and cytokine production.	Provides general immunological context to place OSCC TAMs within the broader iron–immune axis and to discuss how systemic iron status, such as anemia, iron supplementation, might impact oral tumor immunity.
Gaetano, C et al., (2010) (215)	Foundational work on iron and macrophage polarization	Demonstrates that classically activated macrophages adopt an iron-retentive phenotype, whereas alternatively activated macrophages favor iron export, and links these iron programs to distinct inflammatory vs reparative roles.	Provides historical and experimental evidence for the iron-retentive M1 vs iron-exporting M2 paradigm that can be applied to hypothesize iron-related functions of TAM subsets in OSCC lesions.
Miguel P Soares et al., (2016) (216)	Bidirectional relationship between macrophages and iron metabolism	Summarizes macrophage roles in systemic iron turnover(erythrophagocytosis, recycling), their response to hepcidin–ferroportin signaling, and how iron status feeds back on macrophage effector programs.	Provides a mechanistic background on systemic iron control (hepcidin–ferroportin axis) that may influence OSCC via chronic inflammation, anemia of chronic disease, or iron supplementation, and hence indirectly shape oral TAM polarization.
Feng Zhao et al., (2025) (217)	Macrophage metabolism in disease microenvironments	Reviews metabolic pathways (including iron-related processes) that govern macrophage polarization in tumor and inflammatory microenvironments, and discusses therapeutic strategies targeting these pathways to improve immunotherapy.	Provides a translational bridge for OSCC, highlighting how targeting iron-linked metabolic programs in TAMs may synergize with immune checkpoint inhibition or chemoradiotherapy.

### 10.2 Therapeutic challenges and future perspectives

Although strategies targeting iron metabolism—including iron chelators, modulators of iron-related pathways, ferroptosis-based approaches, and nanocarrier delivery systems—have demonstrated potential in preclinical studies, their clinical translation remains challenging. These challenges include insufficient targeting specificity, systemic toxicity risks (e.g., anemia with iron chelation), patient heterogeneity in iron status, and the context-dependent role of ferroptosis in cancer biology. Future research should focus on developing biomarker-guided patient stratification strategies based on iron metabolism–related markers (e.g., ferritin, transferrin receptor, ferroportin, and ferroptosis-associated gene signatures). Additionally, optimizing oral cavity–specific delivery systems and exploring combinatorial strategies integrating iron modulation with immunotherapy and targeted therapies will be critical. Emerging technologies such as single-cell sequencing and spatial transcriptomics may further elucidate iron metabolism dynamics within macrophage subpopulations and tumor niches. These approaches could enable more precise targeting of the iron–macrophage axis and support the development of personalized therapeutic strategies in OSCC. If validated in OSCC-specific clinical models, the iron-macrophage axis may emerge as a pharmacologically actionable target to enhance immunotherapy responses and improve outcomes for patients with this therapeutically challenging disease.
